# Multispectral imaging and automated analysis for quantifying grain quality to reveal known and potential novel alleles affecting grain traits in wheat

**DOI:** 10.3389/fpls.2025.1735309

**Published:** 2026-01-02

**Authors:** Jie Dai, Daiki Abe, Zhengjie Wen, Yuyi Li, Hongyan Li, Jinlong Huang, Phil Howell, Robert Jackson, Ji Zhou

**Affiliations:** 1 College of Engineering, Academy for Advanced Interdisciplinary Studies, Plant Phenomics Research Centre, Nanjing Agricultural University, Nanjing, China; 2 Data Sciences Department, National Institute of Agricultural Botany (NIAB), Crop Science Centre (CSC), Cambridge, United Kingdom; 3 Department of Plant Sciences, Crop Science Centre (CSC), University of Cambridge, Cambridge, United Kingdom

**Keywords:** GWAS, multispectral seed imaging, seed morphologies, seed quality, spectral analysis, wheat

## Abstract

To accelerate the pace of wheat (*Triticum aestivum* L.) improvement worldwide, desired seed-level characteristics and seed quality receive a growing attention as they directly impact early seedling establishment, seed longevity, and grain quality. Nevertheless, the throughput and accuracy of seed-level phenotyping and analysis have become a key limiting factor in this research domain, requiring new solutions to relieve this bottleneck. In this study, we first combined automated multispectral seed imaging (MSI; i.e. the VideometerLab 4 and Autofeeder systems) with a variety of machine learning and computer vision techniques to establish a high-throughput pipeline to analyse wheat seeds. Then, using 493 lines selected from the NIAB Diverse MAGIC (NDM) population, we applied the pipeline to segment individual seeds from MSI seed-lot images. This enabled us to perform seed-level measurement of sixteen morphological (e.g. seed size, length, width, and roundness) and spectral traits, ranging from ultraviolet (i.e. 375 nm, correlating with crude protein) to near-infrared (e.g. 975 nm, for assessing water content) wavelengths. After verifying these seed quality related traits (R2 ≥ 0.949; p < 0.001), we applied genome-wide association studies (GWAS) to link the computationally derived traits to genetic loci and identified eleven significant loci. Some of the loci were previously reported, with two unknown loci valuable for further assessment. Taken together, we believe this integrated MSI analysis pipeline provides a powerful solution for seed research and crop improvement in wheat, enabling us to bridge MSI, seed-level analysis, and genetic mapping to assess seed morphology, seed quality, and their underlying genetic architectures effectively.

## Introduction

Bread wheat (*Triticum aestivum* L.) contributes to one-fifth of daily calories and protein in many nations, making it a crucial staple crop to ensure global food security (Langridge et al., 2022). Due to a rapidly changing climate, the global wheat supply is increasingly threatened, which requires us to ensure wheat production under different growing conditions (Shiferaw et al., 2013; FAO et al., 2023). One approach to tackle this challenge is to accelerate breeding, so that crop improvement can keep up with the pace of current climatic and agronomic changes (Tester and Langridge, 2010). Hence, innovative methods such as genomic selection, CRISPR-Cas9 gene editing, and high-throughput phenotyping are popularly being utilized in recent years, which facilitate breeders and plant researchers to develop climate-resilient crop varieties with aims of enhancing yield production and resource use efficiency (Jaganathan et al., 2018; Cobb et al., 2019).

As a critical factor for plant growth and development, seed quality closely connects with enhanced agronomic performance, including favorite physical attributes (e.g. freedom from damage, disease, and weed seeds), preferred physiological properties (i.e. high and reliable germination and emergence), desired genetic constitution (e.g. cultivar purity and yield potential), and pathological health such as absence of disease-causing microorganisms (Matthews et al., 2012; Domergue et al., 2019). Hence, quality-related seed research is one of the most important agronomic studies as they determine plants’ early biotic and abiotic stress resistance and thus the best possible early growth and development (Peng et al., 2022). Furthermore, as seed quality and seed vigor are often closely connected (Domergue et al., 2019), the assessment of seed quality not only can facilitate us to forecast crop establishment in the field, but also can help us evaluate seed longevity under diverse growing conditions (Peng et al., 2022; Reed et al., 2022). Additionally, seed quality impacts post-harvest seed processing, and seed-level morphological and color features are connected with consumer acceptability due to taste and market preferences. For example, grain size can affect milling yield compared to other factors (Marshall et al., 1986), whereas grain color is not only associated with dormancy but also with different consumer preferences (Kläsener et al., 2019; Mares and Himi, 2021; Afonnikova et al., 2024).

Still, traditional manual assessments remain limited when measuring seed-level traits to evaluate seed quality, which are predominantly destructive, time-consuming, and labor-intensive, making seed quality research inadequate for the rapid, non-destructive, and scalable demands in modern breeding and agricultural production (Elmasry et al., 2019; Shi et al., 2024). For example, commercial devices such as MARViN seed analyzer (MARViTECH GmbH, Wittenburg, Germany) was one of the first and widely adopted instruments used for assessing seed morphological features through vision-based analysis together with the measurement of key yield components (e.g. thousand grain weight, TGW). Recently, many other research-focused seed phenotyping and analytic toolkits were introduced using red-green-blue (RGB) sensors, including: (1) *PhenoSeeder* designed to analyze morphological and color traits (Jahnke et al., 2016); (2) *Germinator* utilized color and contrast features of seed coat and radicle to identify germination status in Arabidopsis (Joosen et al., 2010); (3) *SeedGerm* employed supervised machine learning (ML) to quantify morphological features of seed germination, quantifying cumulative germination rates to enable genetic analysis in Brassica (Colmer et al., 2020); and (4) *SeedGerm-VIG* combined a range of deep learning (DL) and computer vision (CV) techniques to categorize seed vigor (i.e. germination speed and uniformity) in cereals (Dai et al., 2025).

Besides RGB-based seed analysis, spectral channels combined with seed morphological (e.g. size, shape), seed coat color, and multispectral properties are employed to characterize seed quality and estimate biochemical indicators (Shi et al., 2024). The key spectral bandwidths identified in these studies were used to indicate surface and internal substances of cereal seeds. For example: (1) 970 nm was reported to be correlated with water content (Hernandez et al., 2015), (2) 540 and 630 nm for starch (Ma and Deng, 2017), (3) 280 nm for crude protein (Jones and Bridgeman, 2019), (4) 940 nm for plant fat (Sendin et al., 2018), and (5) 642 and 662 nm for chlorophyII (Zhang et al., 2018). The above research drives the applications of multispectral seed imaging (MSI) in seed quality assessment, making the evaluation nondestructive, quantifiable, and reproducible. Nevertheless, it is noticeable that spectral evaluation of seed lots also associates with challenges in throughput, accuracy, and automation.

In this study, we developed an automated and high-throughput analysis pipeline to automate the assessment of seed quality in wheat. Using seed samples collated from replicated trials of 493 lines selected from the NIAB Diverse MAGIC (NDM) population, we first performed MSI of over 70,000 wheat seeds (n = 493 genotypes; 986 seed lots) using the VideometerLab4 and Autofeeder systems. Then, we combined diverse CV- and ML-based techniques (e.g. watershed algorithm, convex hull, and corner detection) to automate seed-level segmentation in MSI images, resulting in eight morphological traits (e.g. seed size, length, width, and roundness) and eight spectral traits, covering from 375 nm (ultraviolet, UV) to 975 nm (near-infrared, NIR) wavelengths. Finally, genome-wide association studies (GWAS) analysis was performed to link seed-level phenotypic variations (i.e. morphological and spectral characteristics) with genetic loci on the chromosome, some of which were reported previously but through many years of study, others were unknown and will require further validation. In conclusion, our work presents a scalable and data-driven framework for large-scale evaluation of wheat seeds, bridging imaging, analysis, and genetics to make effective seed quality assessment, which is valuable for seed-focused crop improvement.

## Materials and methods

### Plant materials and seed production

The plant materials used were selected from the 16-founder NDM population, which consists of 493 wheat lines (**Supplementary Table S1**) with known morphological and grain quality differences, including protein content and thousand grain weight (Scott et al., 2021). After 16-way crosses, agronomic traits of the selected NDM lines were genetically stable and no longer segregating. The lines were drilled in 4-m^2^ (2 × 2 m) plots at NIAB’s Hinxton Big Common trial field (52°09′N, 0°18′E) in early October, 2023, with a row spacing of 30 cm and a sowing density of 1.6 million plants per hectare (ha) (Scott et al., 2021). Plants were managed following standard husbandry practices, including appropriate agronomic inputs such as fertilizers, pest, and fungicide controls according to local conditions. MSI was commonly conducted within three months after harvest, so that we could maximize seed viability and physiological integrity when assessing seed quality (Sano et al., 2016). Before MSI, all seed lots were stored in fridges at ~10°C, with a stable humidity of 65%.

### Multispectral seed imaging

**Figure 1:**
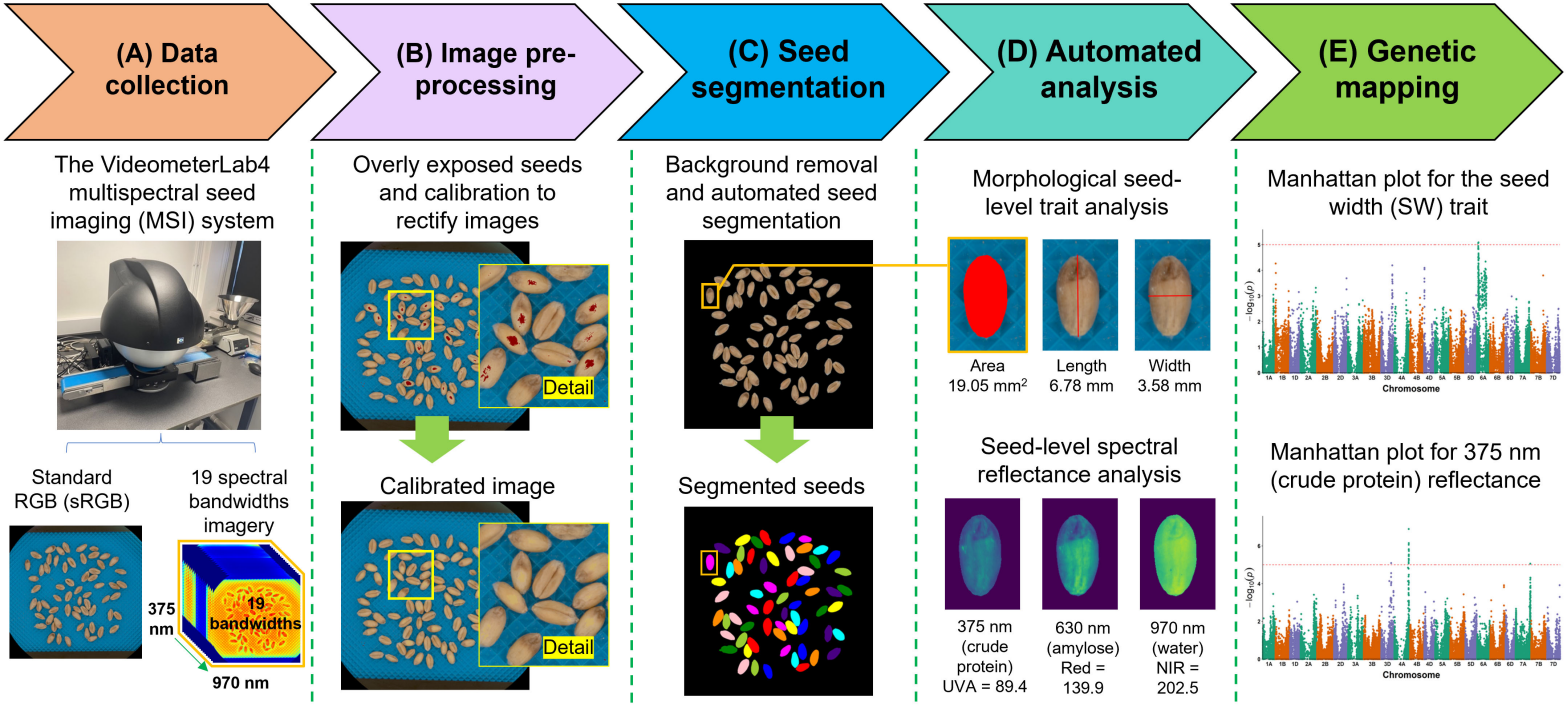
The overall workflow of the study, including multispectral seed imaging, pre-processing, trait analysis, and genetic mapping through GWAS. **(A)** The multispectral seed imaging hardware system and acquired imagery. **(B)** Image pre-processing that includes calibration of overly exposed seed regions. **(C)** The segmentation of seed lot images. **(D)** Seed-level morphological and spectral analysis. **(E)** Two examples of GWAS analysis results.

MSI was performed using the VideometerLab4 device and the Autofeeder system (Videometer A/S, Denmark) (França-Silva et al., 2022), which acquired both standard red-green-blue color space images (sRGB) and MSI datasets (in hips format) covering 19 spectral bandwidths, from 375 nm (ultraviolet, UV) to 970 nm (near infrared, NIR; **Figure 1A**). Due to the relatively low spectral power provided by the Videometer system, it is important to note that the measured reflectance traits were largely based on substances in the out layers of seeds. For multispectral images, 60~80 seeds per seedlot (two seedlots per line) were randomly laid on the Autofeeder’s blue-colored conveyor belt, with 1.5 s for exposure and image acquisition. Roughly, the system is capable of collecting imagery from 3,000 seeds per hour (40–50 seed lots) and over 12,000 seeds per day. Image resolution was kept at 2,192 × 2,192 pixels. A total of 94.1 GB sRGB and MSI data were collected between October and December 2024.

### Image pre-processing and calibration

Before seed-level trait analysis, image pre-processing was carried out to standardize image color and contrast collected from the MSI system, including the rectification of overly exposed seed images. The overexposed regions (i.e. dark red colored pixels; **Figure 1B**, upper) were first identified based on the *a* channel of the CIE L*a*b* color space (Schanda, 2007) using a local adaptive thresholding algorithm (Gonzalez and Woods, 2002). Then, R, G, B values were computed based on the RGB color space in properly exposed regions (i.e. regions without dark red pixels within seeds) to calibrate overexposed regions (**Figure 1B**; lower). To ensure that only seed-level RGB color values were used for calibrating overexposed regions, the grayscale image acquired for 780 nm bandwidth (i.e. between red edge and NIR) was employed, followed by the local adaptive thresholding to remove blue-colored background (i.e. conveyor belt of the Autofeeder system).

### Automated seed segmentation

**Figure 2:**
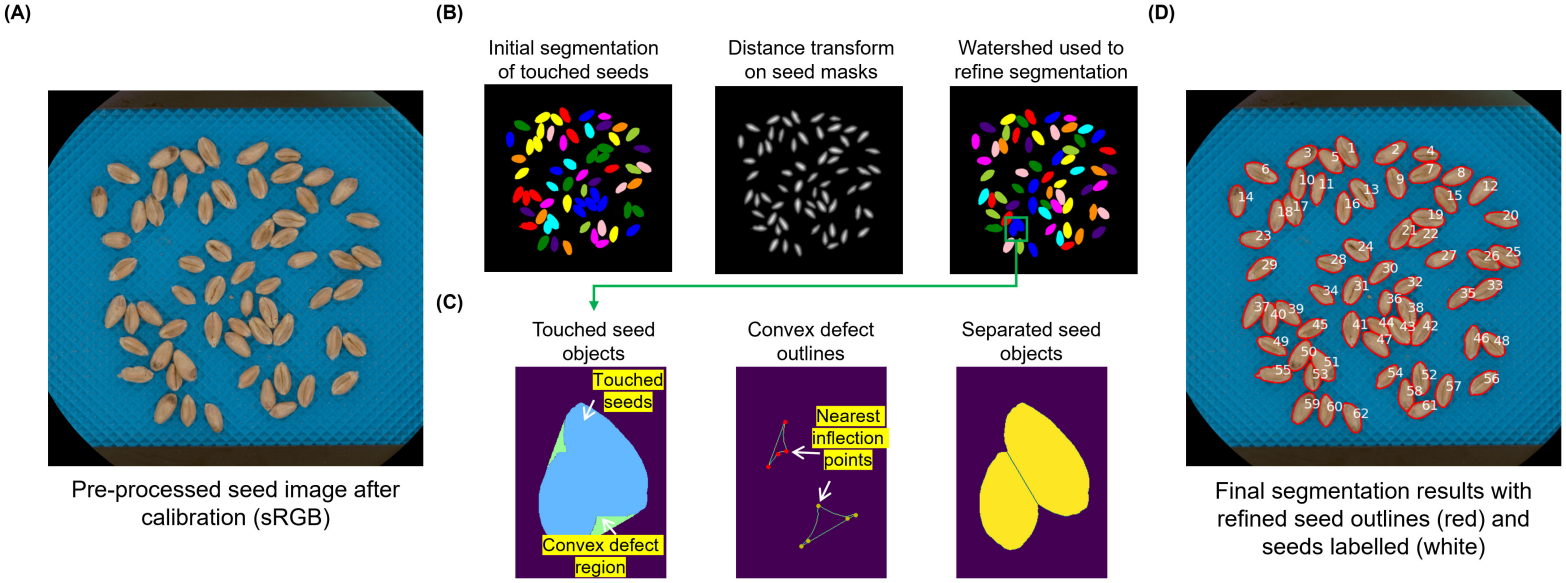
The algorithmic workflow for automated seed segmentation. **(A)** Pre-processed seed image after calibration. **(B)** The application of Euclidean distance transform and watershed algorithm to refine seed object segmentation. **(C)** Segmentation of touched seeds unseparated by the watershed algorithm using the nearest inflection points identified by Harris & Stephens corner detection algorithm on the contours of convex defect regions. **(D)** Final segmentation result, with refined seed outlines (red) and seeds labelled (white).

To automate seed segmentation for seed-level morphological and spectral trait analysis (**Figure 1C**), we first removed blue-colored image background based on 780-nm grayscale images. As the initial seed masks generated contained both single and touched seeds (i.e. seeds whose outlines are connected or overlapped; **Figure 1C**, lower), we developed an object segmentation pipeline (**Figure 2**), including: (1) using the Euclidean distance transform (Fabbri et al., 2008) of the initial seed masks, followed by the use of the watershed algorithm (Meyer, 1992) to refine seed object segmentation (**Figures 2A, B**); (2) because the jagged object edges could affect the accuracy of seed-level morphological analysis, skeletons of these edges and their endpoints were utilized to create lines to connect endpoints (**Supplementary Figure S1**); (3) for seed objects remained touched, a convex hull based method was developed to define convex defect regions (Haria et al., 2017), followed by the identification of nearest inflexion points of contours derived from the convex defect regions using the Harris & Stephens corner detection algorithm (Harris and Stephens, 1988), leading to the formation of lines between the closest points to separate touched seeds (**Figure 2C**); and (4) finally, the convex defect method was also applied to correct over-segmented seeds based on seed-level length and area (**Supplementary Figure S2**). The final seed segmentation results were labelled (white), with recognized seeds outlines (red) in final images (**Figure 2D**). Also, seed experts used the processed images to verify segmentation results, as well as to produce ground-truthing based on seed-level morphological and spectral measures.

### Seed-level trait analysis

Utilizing the finalized seed masks, we first applied diverse vision-based methods to measure a range of key morphological traits reported previously (Tanabata et al., 2012; Colmer et al., 2020), including seed area, convex area, length, width, perimeter, length/width ratio (LWR), eccentricity, and roundness (**Figure 1D**; upper). The algorithmic approaches and software implementation of these measures have been described previously (Colmer et al., 2020). Besides morphological features, we also quantified seed-level spectral reflectance based on mean values of the 19 bandwidths’ grayscale images acquired by the Videometer device (**Figure 1D**; lower), including 375 nm (UV), 405 nm (violet), 435 nm (indigo), 450 nm (blue), 470 nm (blue), 505 nm (cyan), 525 nm (green), 570 nm (yellow), 590 nm (amber), 630 nm (red), 645 nm (red), 660 nm (red), 700 nm (red), 780 nm (deep red), 850 nm (NIR), 870 nm (NIR), 890 nm (NIR), 940 nm (NIR), to 970 nm (NIR).

According to previous studies, some of the 19 spectral bandwidths collected by the Videometer device are related to surface or internal biochemical substances for hibiscus, wheat, and maize seeds (**Figure 1D**; lower). **Table 1** summarizes key bandwidths reported previously, based on which we chose to utilize eight bandwidths due to their biological relevance to seed quality (i.e. the selected bandwidths are close to previously reported ones), including 375 nm, 450 nm, 525 nm, 630 nm, 645 nm, 660 nm, 940 nm, and 970 nm (Hernandez et al., 2015; Ma and Deng, 2017; Sendin et al., 2018; Zhang et al., 2018; Jones and Bridgeman, 2019).

**Table 1:** Key spectral bandwidths reported previously related to biochemical substances in diverse plant seeds.

Videometer band	Substance	Known bandwidth	References	Crop species
375 nm (UV)	Crude protein	~280 nm	(Jones and Bridgeman, 2019)	Hibiscus
450 nm (Blue)	Carotenoids	448 nm	(Zhang et al., 2018)	Wheat
525 nm (Green)	Amylopectin	540 nm	(Ma and Deng, 2017)	Wheat
630 nm (Red)	Amylose	630 nm		Wheat
645 nm (Red)	ChlorophyII B	642 nm	(Zhang et al., 2018)	Wheat
660 nm (Red)	ChlorophyII A	662 nm		Wheat
940 nm (NIR)	Fat	940 nm	(Sendin et al., 2018)	Maize
970 nm (NIR)	Water	970 nm	(Hernandez et al., 2015)	Wheat

### Genome-wide association study

**Figure 3:**
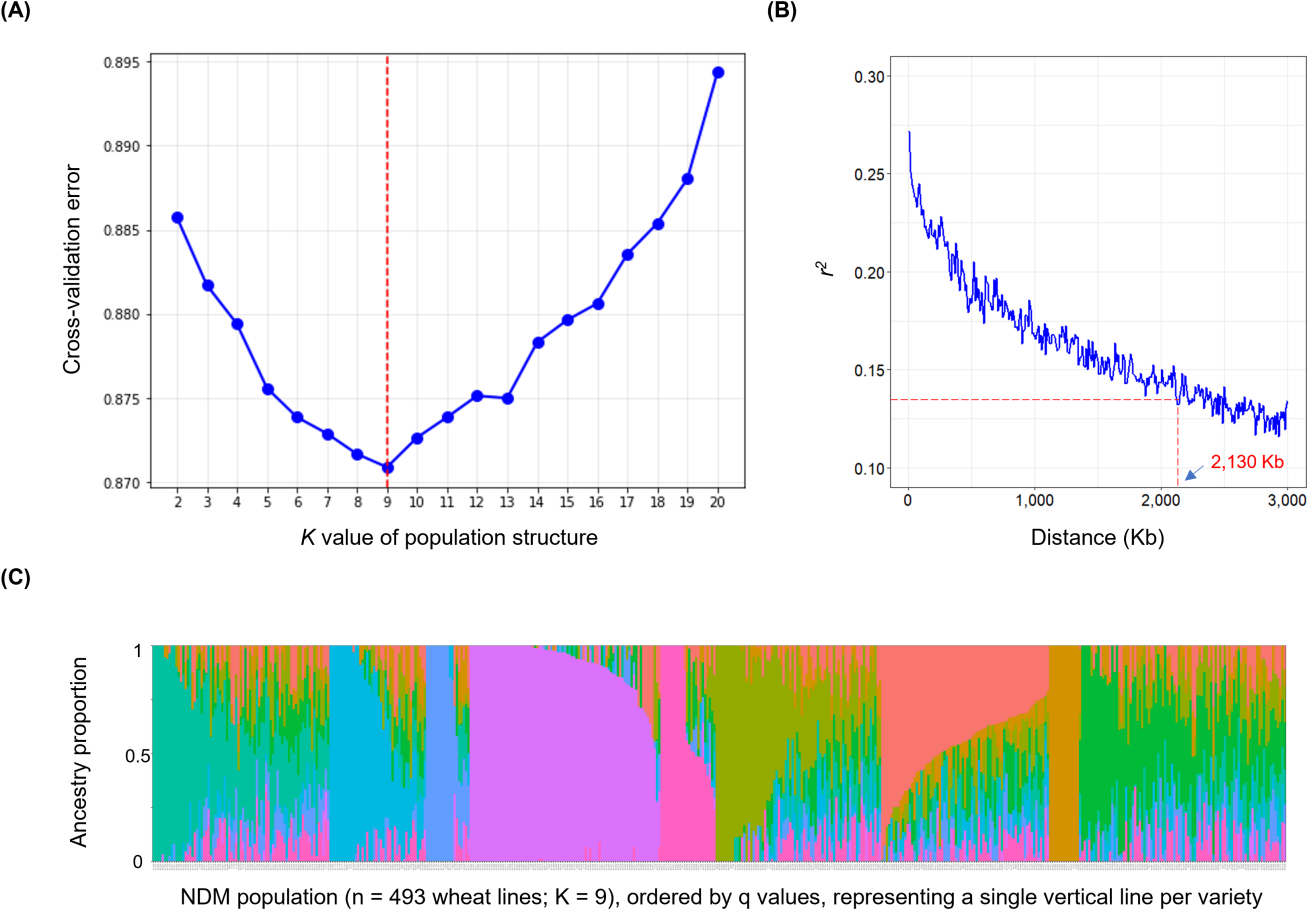
*K* value of population structure and decay of linkage disequilibrium in the 493 NDM wheat lines. **(A)** The population structure analysis; **(B)** The LD decay distance of the 493 lines; **(C)** The population structure inferred by ADMIXTURE analysis (K = 9) using the lines, where different colors represent different subgroups.

Single nucleotide polymorphism (SNP) loci data of the NDM population were retrieved from a previous study (Scott et al., 2021). A total of 55,067 SNPs with minor allele frequency (MAF) > 0.05 were used in GWAS analysis (**Figure 1E**), which was conducted by GCTA (v1.94.1; Yang et al., 2011) using a mixed linear model (MLM), with a population structure (**Figure 3A**) inferred by ADMIXTURE (v1.3.0) (Alexander et al., 2009) and Kinship matrix generated by PLINK (v1.90b6.21) (Chang et al., 2015). SNPs with a *P* value below the widely accepted threshold of 1e-5 (Pang et al., 2020; Eltaher et al., 2021) were retained and then referenced to the Chinese spring reference genome (IWGSC Ref Seq v1.0) (IWGSC, 2018), as well as the genome annotation file (Ref Seq Annotation v1.2). The linkage disequilibrium (LD) decay distance of the NDM population (**Figure 3B**) was estimated by PopLDdecay (v3.43) (Zhang et al., 2019) and determined to be ~2,130 kb (Huang et al., 2010). Bedtools (v2.18) (Quinlan and Hall, 2010) was used to obtain potential candidate genes from significant SNPs, within up- and down-stream 2,130 kb regions, which were compared with known QTL regions or examined according to their functions. The visualization of the population structure inferred by ADMIXTURE analysis (K = 9) is given (**Figure 3C**, **Supplementary Figure S3**).

### Manual scoring and statistical analysis

After seed-level trait analysis, the scale was measured to convert pixels into metric unit (i.e. millimeters, mm). To verify results of seed segmentation and trait analysis, a total of 100 seed lots were randomly selected from the MSI image set, followed by manual scoring of four morphological traits (i.e. seed area, seed length, seed width, and seed perimeter) and all eight spectral traits. The manual measures of seed-level morphologies and spectral features were conducted using ImageJ (Schneider et al., 2012), based on seeds randomly selected from the seed lots. The Kolmogorov-Smirnov test (Massey, 1951) was performed to determine whether traits followed a normal distribution, while correlation analysis was performed using the Pearson correlation coefficient (Pearson, 1895) and *P*-value after removing the outliers. The broad-sense heritability was estimated as the ratio of total genetic variance to total phenotypic variation, with variance components obtained using a linear mixed model (Nyquist and Baker, 1991). To evaluate the accuracy of detected seed masks for subsequent object segmentation, mean intersection over union (mIoU) was used to compare manual and computational seed masks.

### Software implementation

When developing the analytical framework, a Windows 10 workstation (16 GB memory, Nvidia GTX 1660Ti GPU, and Intel Core i7-10700F CPU) was used. We employed several key open-source scientific libraries for software implementation, including the scientific data processing library ‘SciPy’ (Virtanen et al., 2020) and the image processing library ‘Scikit-Image’ (van Der Walt et al., 2014). All figures except GWAS related figures were plotted using the Python libraries ‘matplotlib’ (Hunter, 2007) and ‘seaborn’ (Waskom, 2021). R packages ‘qqman’ and ‘ggplot2’ (Wickham, 2016) were used to produce GWAS related figures. Statistical analysis was also performed using the ‘SciPy’ and ‘Statsmodel’ libraries (Seabold and Perktold, 2010).

## Results

### The automated pipeline for seed analysis

After establishing the automated analytic pipeline (**Figures 1A-D**), we processed 493 NDM wheat genotypes (2 replicates; 986 seed lots, over 70,000 seeds) using the pipeline, covering from image pre-processing (e.g. color calibration and filling overexposed seed regions), seed segmentation with touched seeds divided using convex defect regions and the connection of nearest inflection points, and seed-level trait analysis based on morphological and spectral properties of the MSI images. Then, we assessed the automated analysis result of seed segmentation against manually counted seed number and seed object regions from 100 seed lots, obtaining a highly significant positive correlation (R^2^ = 0.996, *RMSE* = 0.656, *P*-value < 0.001; mIoU = 0.947; **Supplementary Figure S4**). Based on the segmented seed objects, we measured 16 seed-level morphological and spectral traits from the MSI images using our pipeline.

### Validation of computationally derived traits selected from NDM

To validate the computationally derived morphological and spectral traits, we utilized the 12 manually assessed traits to perform correlation analyses, including seed area (n = 1,184 seeds; R^2^ = 0.980, *P*-value < 0.001, RMSE = 2.07 mm^2^), seed length (n = 1,184 seeds; R^2^ = 0.952, *P*-value < 0.001, RMSE = 0.38 mm), seed width (n = 1,184 seeds; R^2^ = 0.974, *P*-value < 0.001, RMSE = 0.23 mm), seed perimeter (n = 1,184 seeds; R^2^ = 0.949, *P*-value < 0.001, RMSE = 1.11 mm), 375-nm reflectance trait (n = 1,184 seeds; R^2^ = 0.993, *P*-value < 0.001, RMSE = 0.23), 450-nm reflectance trait (n = 1,184 seeds; R^2^ = 0.994, *P*-value < 0.001, RMSE = 0.46), 525-nm reflectance trait (n = 1,184 seeds; R^2^ = 0.995, *P*-value < 0.001, RMSE = 1.02), 630-nm reflectance trait (n = 1,184 seeds; R^2^ = 0.984, *P*-value < 0.001, RMSE = 2.31), 645-nm reflectance trait (n = 1,184 seeds; R^2^ = 0.980, *P*-value < 0.001, RMSE = 2.70), 660-nm reflectance trait (n = 1,184 seeds; R^2^ = 0.977, *P*-value < 0.001, RMSE = 2.86), 940-nm reflectance trait (n = 1,184 seeds; R^2^ = 0.984, *P*-value < 0.001, RMSE = 1.54), and 970-nm reflectance trait (n = 1,184 seeds; R^2^ = 0.985, *P*-value < 0.001, RMSE = 1.46).

**Figure 4:**
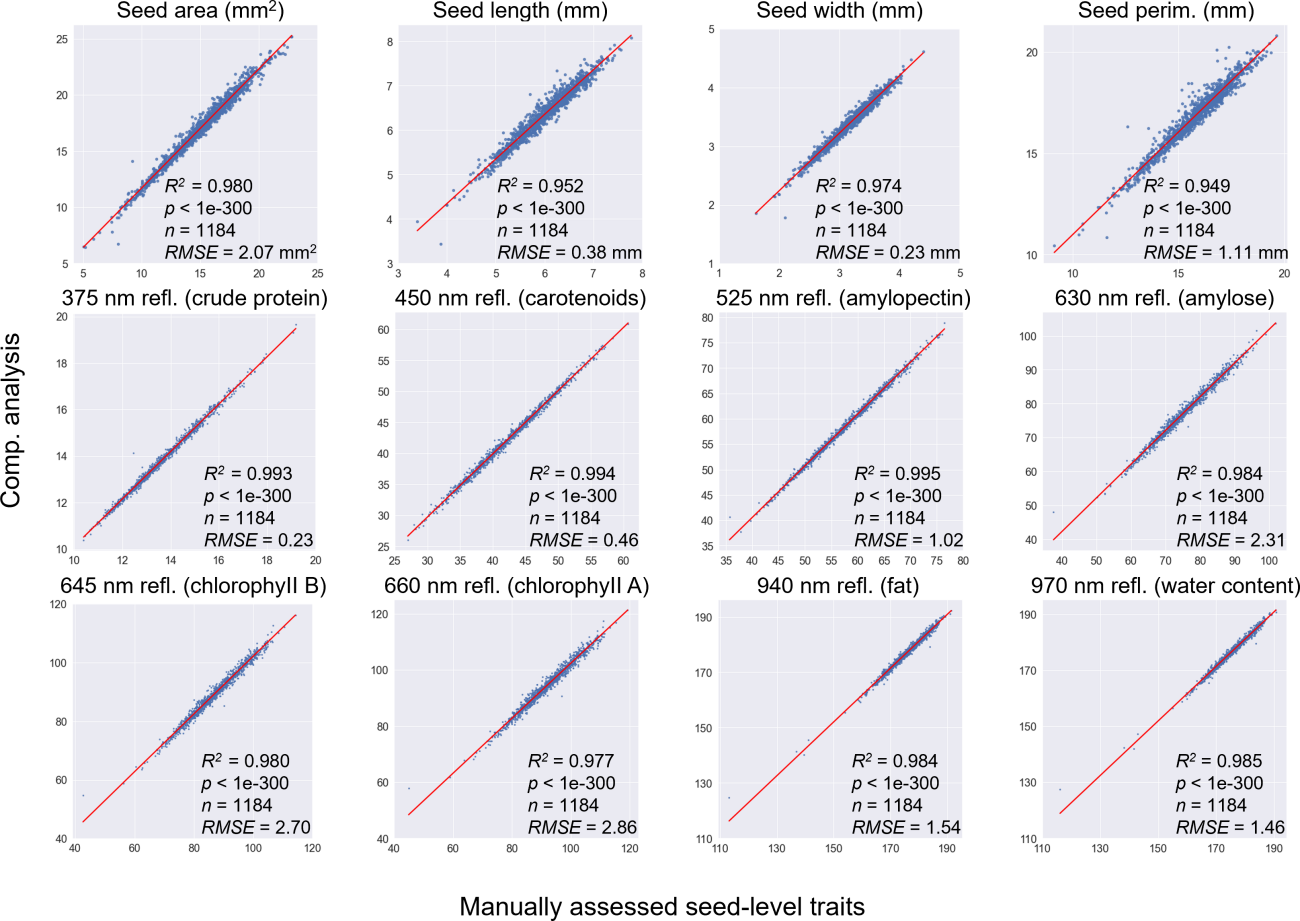
Correlation analyses performed to compare computationally derived and manually measured traits, including seed area, seed length, seed width, seed perimeter, and spectral reflectance traits based on 375, 450, 525, 630, 645, 660, 940, and 970 nm MSI grayscale images.

According to the analyses (**Figure 4**), significant positive correlations were observed, indicating the reliability of the computational traits quantified by the automated pipeline. In order to reveal the genetic characteristics of quality-related traits, descriptive analysis of the traits revealed considerable variation among the NDM genotypes, as well as the broad-sense heritability (**Supplementary Table S2**). Also, most of the traits followed a normal distribution except seed LWR, roundness, 630-nm and 645-nm spectral reflectance traits (**Supplementary Figure S5**).

### GWAS using seed-level morphological traits

**Figure 5:**
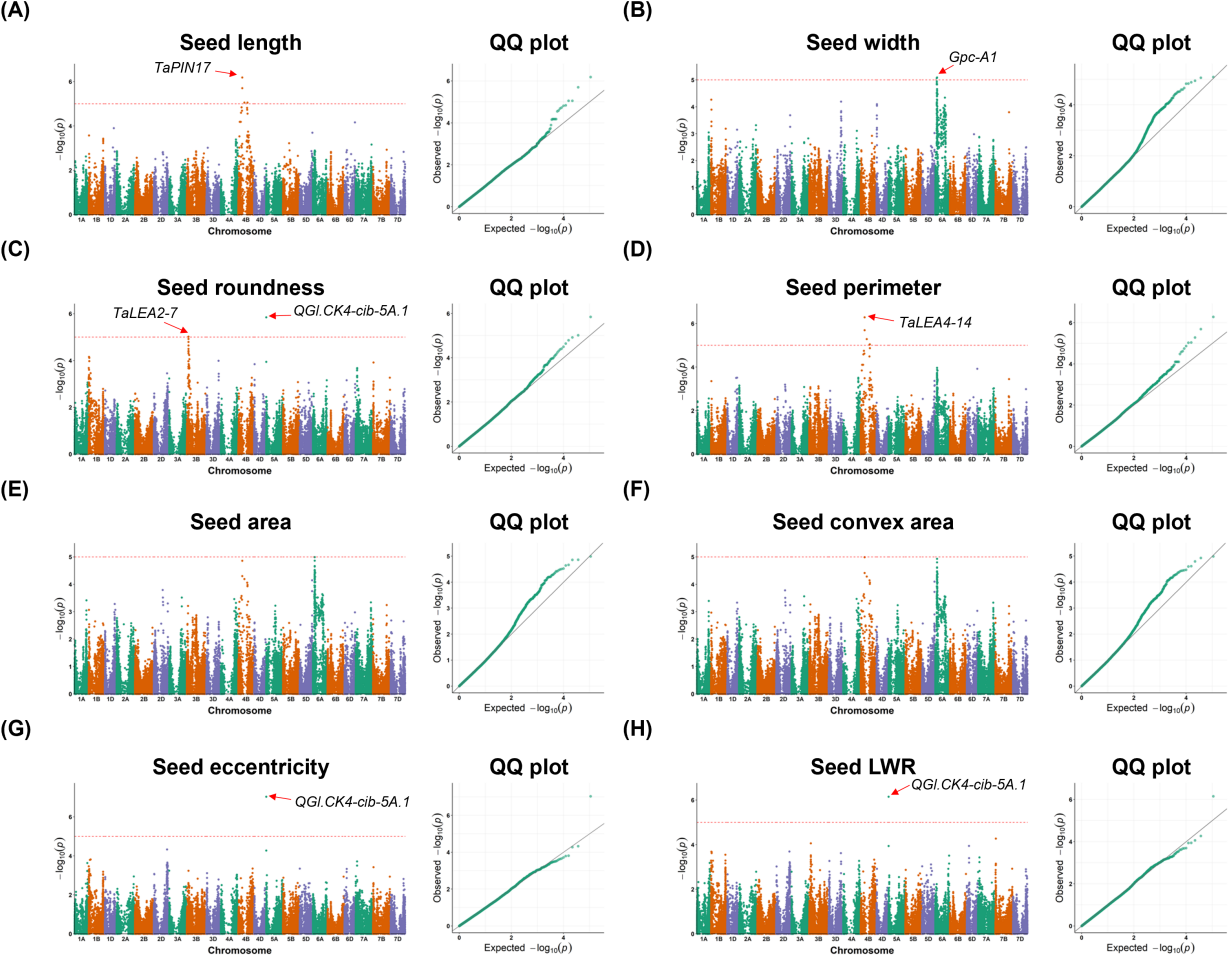
Manhattan plots and quantile-quantile (QQ) plots for morphological traits subjected to a genome-wide association studies (GWAS) of 493 wheat NDM lines. The significance threshold is shown by the horizontal red dotted line. Known genes or QTLs that co-locate with significant loci are indicated by red arrow. **(A-H)** Manhattan plots and QQ plots are provided for morphological traits such as seed length, width, roundness, perimeter, area, convex area, eccentricity, and length/width ratio (LWR).

We further verified the biological relevance of the computationally derived traits using GWAS analysis, identifying significant genetic loci using morphological and spectral variations of the 493 wheat lines (**Figure 5**). We identified several significant SNPs associated with quality-related morphological traits and presented them in the Manhattan plot and Quantile-Quantile (QQ) plot, with a red dotted line indicating the threshold for the genome-wide significant *P*-values. For example, using the seed length trait, we located a strong signal on chromosome 4B (-1og_10_*P* = 6.18; **Figure 5A**) 1,571.0 kb away from *TaPIN17*, which is known for spike development (Kumar et al., 2021; Gong et al., 2022). Similarly, using the seed width trait, a strongest signal on chromosome 6A (-1og_10_*P* = 5.09, indicated with a red arrow; **Figure 5B**) was identified, 789.8 kb from *Gpc-A1* that regulates the contents of grain protein, zinc, and iron (Uauy et al., 2006). Other seed morphological traits such as seed roundness and perimeter were used to identify significant SNPs (-1og_10_*P* = 5.02 and -1og_10_*P* = 5.03, respectively) associated with late embryogenesis-abundant (LEA) genes that play an important role during seed maturation (Liu et al., 2019), whereas seed roundness, eccentricity and length/width ratio (LWR) were employed to locate SNPs associated with grain length quantitative trait locus (QTL), *QGl.CK4-cib-5A.1* (Li et al., 2025). **Table 2** lists all the significant signals identified by GWAS based on seed-level morphological traits.

**Table 2:** Significant loci identified by GWAS using seed-level morphological traits relevant to seed quality (n = 493 wheat NDM lines).

Trait	Chr.	Position	-log_10_*P*	Distance (kb)	Candidate gene	Gene symbol	Known QTL
Seed length	4B	172,500,715	6.18	1,571.005	*TraesCS4B02G130100*	*TaPIN17*	
Seed width	6A	77,890,175	5.09	789.814	*TraesCS6A02G108300*	*Gpc-A1*	
Seed LWR	5A	30,375	6.15				*QGl.CK4-cib-5A.1*
Seed roundness	3B	714,01,947	5.02	916.319	*TraesCS3B02G106700*	*TaLEA2-7*	
	5A	30,375	5.84				*QGl.CK4-cib-5A.1*
Seed eccentricity	5A	30,375	7.03				*QGl.CK4-cib-5A.1*
Seed perimeter	4B	387,376,501	5.03	1,750.962	*TraesCS4B02G175600*	*TaLEA4-14*	

### GWAS using seed-level spectral traits

**Figure 6:**
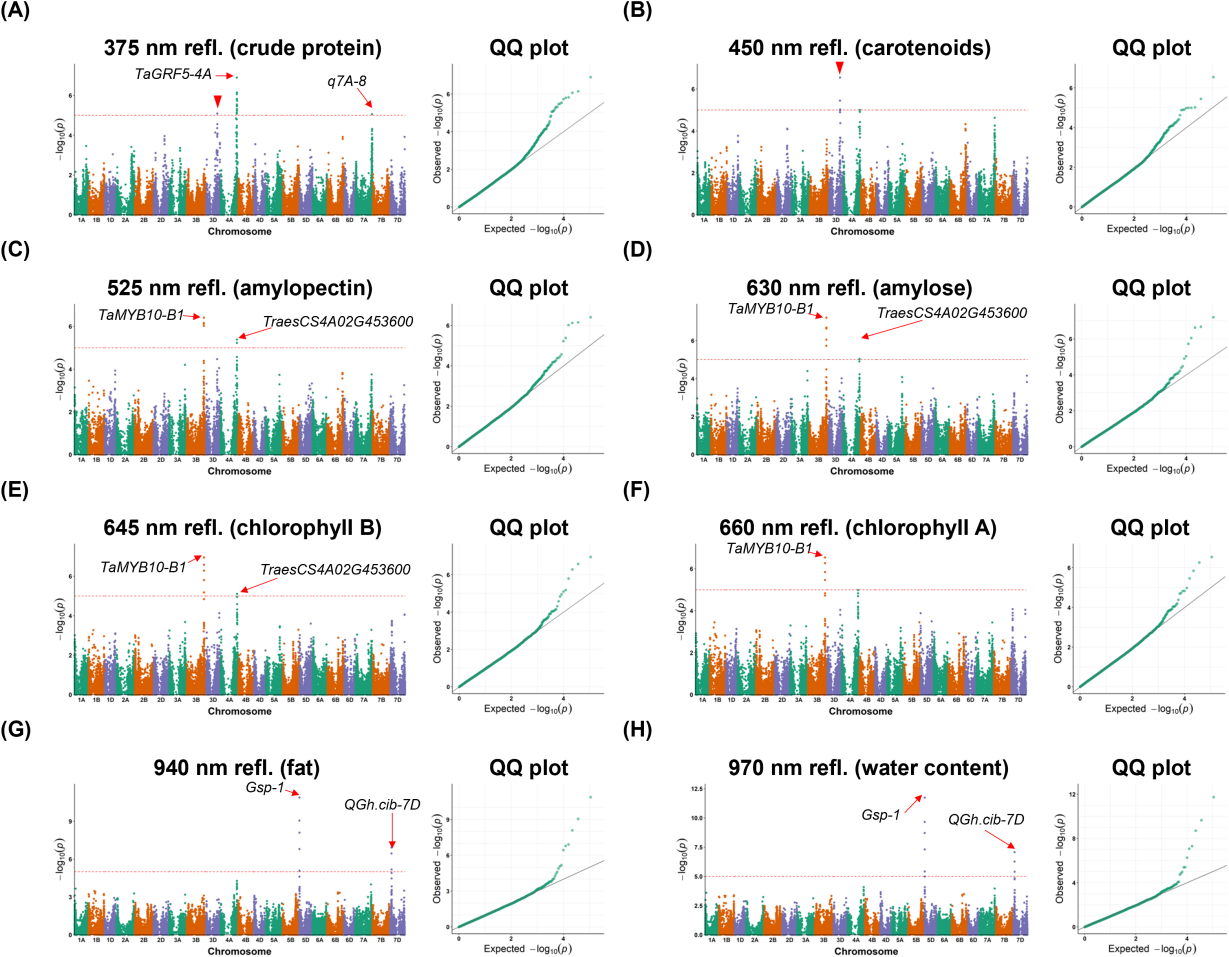
Manhattan plots and quantile-quantile (QQ) plots for spectral traits subjected to a genome-wide association studies (GWAS) of 493 wheat NDM lines. The significance threshold is shown by the horizontal red dotted line. Known genes or QTLs that co-locate with significant loci are indicated by red arrow, whereas unknown loci are indicated by red triangles. **(A-H)** Manhattan plots and QQ plots are given for spectral traits using 375 nm, 450 nm, 525 nm, 630 nm, 645 nm, 660 nm, 940 nm, and 970 nm reflectance, which were reported for correlating with various biochemical substances.

Using spectral traits, a range of significant SNP loci were identified. For example, using the 375 nm reflectance trait, we located a strong signal on chromosome 4A (-1og10P = 6.9; **Figure 6A**), 711.5 kb away from *TaGRF5-4A* that is known for spike development (Yao et al., 2024). Besides, another strong signal on chromosome 7A (-1og10P = 5.06; **Figure 6A**) was identified, associated with dough development related QTL *q7A-8* (Yang et al., 2020). Similarly, by using the green and red spectral bands (i.e. 525 nm, 630 nm, 645 nm, and 660 nm), we associated *TaMYB10-B1* which had been reported to change seed color (Himi and Noda, 2005) and seed storage protein *TraesCS4A02G453600* (Zhao et al., 2023), while the significant SNP loci identified in the NIR bands (i.e. 940 nm, and 970 nm) were associated with grain hardness and endosperm texture, including *Gsp-1* and *QGh.cib-7D* with a close linkage with the *Pin* genes (Bhave and Morris, 2008; Liu et al., 2024). **Table 3** summaries the significant signals for all the spectral traits.

**Table 3:** Significant loci identified through GWAS using seed-level spectral traits relevant to seed quality (n = 493 wheat NDM lines).

Trait	Chr.	Position	-log_10_*P*	Distance (kb)	Candidate gene	Gene symbol	Known QTL
375 nm refl. (crude protein)	3D	482,587,803	5.09		*Unknown*		
	4A	704,798,377	6.9	711.535	*TraesCS4A01G434900*	*TaGRF5-4A*	
	7A	720,641,612	5.06				*q7A-8*
450 nm refl. (carotenoids)	3D	482,587,803	6.54		*Unknown*		
525 nm refl. (amylopectin)	3B	757,916,410	6.16	1.854	*TraesCS3B02G515900*	*TaMYB10-B1*	
	4A	718,422,228	5.22	449.529	*TraesCS4A02G453600*		
630 nm refl. (amylose)	3B	757,916,410	6.68	1.854	*TraesCS3B02G515900*	*TaMYB10-B1*	
	4A	718,422,731	5.03	450.032	*TraesCS4A02G453600*		
645 nm refl. (chlorophyII B)	3B	757,916,410	6.58	1.854	*TraesCS3B02G515900*	*TaMYB10-B1*	
	4A	718,422,731	5.10	450.032	*TraesCS4A02G453600*		
660 nm refl. (chlorophyII A)	3B	757,916,410	6.27	1.854	*TraesCS3B02G515900*	*TaMYB10-B1*	
940 nm refl. (fat)	5D	5,816,064	5.08	18.508	*TraesCS5D02G004000*	*Gsp-1*	
	7D	56,981,157	5.18				*QGh.cib-7D*
970 nm refl. (water)	5D	5,816,064	5.41	18.508	*TraesCS5D02G004000*	*Gsp-1*	
	7D	56,981,157	5.39				*QGh.cib-7D*

## Discussion

As a key agronomic trait, seed quality is key to cereal crop’s early establishment, early stress tolerance, and yield potential (Matthews et al., 2012). Nevertheless, seed-based phenotyping remains a major bottleneck in seed-focused crop improvement as traditional manual assessment is labor-intensive, destructive, limited in throughput (Elmasry et al., 2019). MSI offers a promising solution due to its high-throughput, non-destructive, and insightful features through spectral bandwidths, which is increasingly used by the plant and crop research community (Elmasry et al., 2019). In this study, we first used the Videometer system to collect large-scale MSI datasets from 493 NDM lines, followed by the development of an automatic analytic pipeline to segment seeds from seed-lot images. Using the pipeline, we quantified 16 seed-level morphological and spectral traits, based on which significant SNP loci were identified that associated with known and unknown genes or QTLs that are relevant to seed quality. As a result, we believe that we have made several advances in terms of the development of a data-driven and scalable MSI framework for automated and powerful seed phenotyping, producing seed-level morphological and spectral traits to enable the examination of seed quality related genetic architectures in wheat.

### The automated pipeline for seed-level seed quality analysis

Combining DL and CV has substantially advanced plant seed phenotyping and automated trait analysis in recent years (Kassem, 2025). By integrating watershed algorithm, convex hull method, and the Harris & Stephens corner detection, we developed the analysis pipeline that was capable of effectively segmenting individual seeds from seed-lot images containing hundreds of wheat seeds acquired by the VideometerLab4 platform, with a very high accuracy (mIoU = 0.947; **Supplementary Figure S4**, **Figure 2D**). This integration enabled us to perform accurate measures of morphological and spectral traits at the single-seed level. Moreover, our research demonstrate that this automated pipeline enabled us to perform consistent, large-scale, and objective evaluation of key seed-level traits such as seed morphologies and seed coat color, which are critical for crop improvement and agricultural production (Reed et al., 2022). Traditional seed assessment methods are often slow, labor-intensive, and prone-to-error, limiting their scalability in modern crop breeding. In contrast, automation presented in this study was capable of processing thousands of seed lots (i.e. hundreds of thousands of seeds) within hours’ computation, with reproducible morphological and spectral measurements to signify seed-lot- and seed-level physiological quality and biochemical compositions.

This methodological advance aligns with contemporary directions in seed science and data analytics, which aim to enable near real-time evaluation of morphological, color, and spectral characteristics in large seed populations (Shi et al., 2024; Dai et al., 2025). Moreover, by integrating imaging, CV- and ML-based analytics, the pipeline collects and generates standardized imagery and analytic datasets that can be used to link to genomic and crop production, enhancing the discovery of genetic determinants that control seed quality and relevant early crop performance. Such an approach is poised to accelerate the selection of plant genotypes with desirable attributes (e.g. improved grain size, internal composition, nutritional value, and shelf life), while enabling systematic exploration of genotype-phenotype relationships to drive innovations in seed biology, breeding, and precision agriculture.

### Seed quality evaluation using multispectral seed imaging

As inherently complex traits that integrate multiple aspects of seed biology, seed quality related traits incorporate physical seed features, physiological performance, genetic composition, and pathological health (Reed et al., 2022). These factors collectively determine seed vigor, viability, and overall value of seed lots, influencing not only in-field emergence but also post-harvest seed quality and storage potential. Hence, understanding seed quality requires a powerful framework that can capture both external morphology and internal biochemical composition with high accuracy and reproducibility (Li et al., 2025). We employed MSI to characterize both morphological and spectral attributes of wheat seeds, providing a comprehensive analysis of seed quality-related traits. The approach allowed us to examine seed morphologies (e.g. seed area, convex area, length, width, perimeter, LWR) and spectral characteristics (with 375, 450, 525, 630, 645, 660, 940, and 970 nm bandwidths) that correlate with substances such as pigment concentration, protein, and starch content. These high-dimensional datasets revealed substantial variations across the 493 lines in the NDM resource (**Supplementary Table S2**), illustrating the power of MSI in dissecting seed quality in a population of lines. To verify the accuracy and reliability of the automated analysis, we conducted a comparative analysis between computationally derived and manually scored measurements using 1,184 seeds, resulting in strong significant relationships, with R² ranging from 0.949 to 0.994 for 12 selected traits (**Figure 4**).

In particular, the NDM population used in this study is a random and unselected multi-parental mapping population derived by intercrossing 16 diverse elite wheat founders representative for over 70 years of UK and part of European wheat breeding (1935-2004), which was established following a funnel crossing scheme over four sequential generations (i.e., 2-way, 4-way, 8-way and 16-way) as well as inbreeding and the final extraction of highly recombinant inbred lines (Scott et al., 2021). Hence, the NDM possesses quantitative variations for many traits that have been fixed in the population through historical selection, with above average diversity for North-West European wheat varieties, but less than average diversity for wider European and global wheat collections. Hence, observed diversities in seed morphological and spectral profiles can also be used to reflect the evolutionary and selective decisions imposed during wheat domestication (Gegas et al., 2010). For example, plant breeders preferentially selected for larger and plumper grains with desired seed coat colors (e.g. darker color) over many generations of domestication and breeding, leading to seed-level traits associated with improved yield potential and improved pre-harvest sprouting resistance, respectively (Peng et al., 2011; Afonnikova et al., 2024). This could explain the reasons why traits such as seed length/width, roundness, and reflectance at 630 and 645 nm (within the red spectral regions) deviated from normally distributed patterns in NDM (**Supplementary Figure S5**), highlighting the influence of domestication and manual selection in modern crop improvement in wheat. Collectively, these results indicate that seed quality evaluation introduced here can be used as a powerful tool to offer new opportunities for seed-focused genetic studies and integrative crop improvement programs.

### GWAS-based genetic mapping

Building upon the computationally quantified morphological and spectral traits, we performed GWAS to identify genetic loci associated with seed quality using the 493 NDM wheat lines, integrating reliable and high-resolution phenotypic variations with genomic analysis to demonstrate the potential of automated MSI in bridging the gap between seed quality related morphological and spectral traits and their underlying genetic determinants. The GWAS analysis successfully identified several genes known for regulating seed-level morphology and coat color, including *TaMYB10-B1*, *Gsp-1*, and *Gpc-A1* together with previously reported QTLs such as *QGl.CK4*-*cib*-*5A.1* (Uauy et al., 2006; Bhave and Morris, 2008; Liu et al., 2024; Li et al., 2025). These loci, which historically required many years of manual measurements and evaluations to uncover, were identified in our study through only several months of data collection and analysis. This not only confirms the identified loci align with known regulators of grain pigmentation, protein accumulation, and grain filling, but also demonstrates that the developed framework can produce biologically relevant results while substantially reducing the time required to generate functionally relevant genetic insights.

For the morphological traits, the significant SNP loci were associated with QTLs controlling seed length and shape, as well as with genes linking to grain protein content and seed maturation-related proteins (**Figure 5**, **Table 2**). We also examined whether the significant loci identified in this study overlapped with the major QTLs controlling grain size and shape reported previously (Gegas et al., 2010). By converting marker positions into physical distances (Zhao et al., 2019) with a significant threshold of -log_10_(*P*) ≥ 3, we found that seed eccentricity was associated with major QTL regions located at the ends of chromosomes 2D (between *cfd233* and *wmc41*) and 4B (between *gwm149* and *wmc47*), whereas seed-level LWR and roundness were both linked to the major QTL at the end of chromosome 4B (between *gwm149* and *wmc47*). These findings may be caused by the limited seed-level morphological variations among lines selected, making it difficult to detect all major QTLs associated with seed shape.

Similarly, GWAS results derived from spectral variations identified significant SNPs related to grain hardness, seed coat color, and spike development (**Figure 6**, **Table 3**). It is worth noting that the spectral bands at 525 nm (green), 630 nm (red), 645 nm (red), and 660 nm (red) were associated with *TaMYB10-B1* and *TraesCS4A02G453600*, which may be attributed to the fact that red-color spectral regions are associated with the seed coat color gene *TaMYB10-B1*, whereas the 525 nm (green) band corresponds to the absorption characteristics of chlorophyII a (~660 nm) and chlorophyII b (~645 nm). In addition, spectral bands (940 and 970 nm, both NIR) are associated with genes involved in grain hardness regulation, resulting in the NIR absorption indirectly reflecting the grain substances.

The above identified loci indicate that both types of traits can provide complementary insights, collectively reflecting seed quality through shared and distinct genetic pathways. Notably, several previously unreported SNP loci were also identified, representing novel candidate regions involved in the formation and regulation of seed quality during key growth stages. The discovery is likely to expand our understanding of the genetic architecture underlying seed morphology and composition, providing a foundation for future functional genomics and molecular breeding for improving nutritional value, desired agronomic features, and crop management in wheat.

### Limitations and future developments

This study has made valuable advances in MSI and seed quality assessment. Still, several limitations should be acknowledged, including further improvements in analytics, scalability, and accessibility. For example: (1) presently, GWAS analysis was performed using individual spectral bandwidth, which may constrain the detection of additional novel loci associated with seed quality using multi-band signals; hence, future studies could enhance the resolution of genetic mapping by incorporating multi-band and integrated signals to better capture spectral variations related to biochemical substances for genotype-phenotype associations; (2) relatively high cost of MSI instruments remains a key barrier for wide adoption in seed research and breeding, which could be mitigated by leveraging affordable optical sensors and compact imaging modules based on limited bandwidths identified for seed quality assessment; (3) due to the spectral range of the Videometer system, it is important to note that the measured reflectance traits were largely based on substances in the out layers of seeds; future work could apply Short-Wavelength InfraRed (SWIR) imaging with relatively high spectral power to characterize seed-level substances; (4) since three-dimensional (3D) seed analyses have been applied to quantify wheat, barley, and oat grains (Hughes et al., 2017; Evershed et al., 2024; Plutenko et al., 2025), multi-dimensional features (e.g., RGB, multispectral reflectance, and 3D point clouds) are likely to be valuable to enable high-throughput and more comprehensive characterization of seed-level traits; (5) finally, improving accessibility of trait analysis is critical, which requires new thoughts into software implementation and human-system-interaction that can enable breeders, researchers, and growers to utilize with limited computational background; to address this problem, cloud-based platforms could be considered to bridge the gap between advanced analytics and practical applications. Taken together, these future developments will help us make our work applicability, scalability, and robustness of multispectral imaging-based seed phenotyping, supporting its integration into a wider range of crop species, experimental conditions, and research objectives.

## Data Availability

The original contributions presented in the study are included in the article/Supplementary Material. SNP loci data of the NDM population were obtained from the repository (http://mtweb.cs.ucl.ac.uk/mus/www/MAGICdiverse/MAGIC_diverse_FILES/BASIC_GWAS.tar.gz); testing multi-spectral imagery can be downloaded from the BioImage Archive repository S-BIAD2408 (DOI: 10.6019/S-BIAD2408). Source codes that support the results of this paper is available at https://github.com/The-Zhou-Lab/Videometer_Seed_Imaging_Analytic_Pipeline/releases.

## Glossary

R^2^: Coefficient of determination
CV: computer vision
DL: deep learning
GWAS: genome-wide association studies
ha: hectare
LEA: late embryogenesis-abundant
LWR: length/width ratio
LD: linkage disequilibrium
mIoU: mean intersection over union
MAF: minor allele frequency
MLM: mixed linear model
MSI: multispectral seed imaging
NIAB: national institute of agricultural botany
NIR: near infrared
NDM: NIAB Diverse MAGIC
QQ: quantile-quantile
QTL: quantitative trait locus
RGB: red-green-blue
RMSE: root mean square error
sRGB: standard red-green-blue color space images
SNP: single nucleotide polymorphism
UV: ultraviolet
