# Supplementary material for: Multispectral imaging and automated analysis for quantifying grain quality to reveal known and potential novel alleles affecting grain traits in wheat
Source: Front Plant Sci. 2026 Jan 2;16:1735309. doi: 10.3389/fpls.2025.1735309 (PMC12808467; doi:10.3389/fpls.2025.1735309)
Supplement: Supplementary file 1 [file DataSheet1.docx]

**Supplementary Material**

## **Title:** Multispectral imaging and automated analysis for quantifying grain quality to reveal known and potential novel alleles affecting grain traits in wheat

**Authors:** Jie Dai^1+^, Daiki Abe^2,3+^, Zhenjie Wen^1+^, Yuyi Li^2,3^, Hongyan Li^1^, Jinlong Huang^1^, Phil Howell^2^, Robert Jackson^2,*^, Ji Zhou^1,2,4*^

**Address:**

^1^College of Engineering, Academy for Advanced Interdisciplinary Studies, Plant Phenomics Research Centre, Nanjing Agricultural University, Nanjing 210095, China

^2^Data Sciences Department, National Institute of Agricultural Botany (NIAB), Crop Science Centre (CSC), Cambridge CB3 0LE, United Kingdom

^3^Department of Plant Sciences, Crop Science Centre (CSC), University of Cambridge, Cambridge CB2 3EA, United Kingdom

^4^Present address: State Key Laboratory of Plant Trait Design, CAS Center for Excellence in Molecular Plant Sciences, Shanghai Institute of Plant Physiology and Ecology, Chinese Academy of Sciences (CAS), Shanghai 200032, China

**Corresponding authors:**

Robert.Jackson@niab.com, Ji.Zhou@NJAU.edu.cn or Ji.Zhou@niab.com

**Supplementary Table S1.** The 493 wheat genotypes selected from the NDM population, with 16 seed spectral and morphological traits measured by the automated analysis pipeline.

| **Line** | **375 nm refl.** | **450 nm refl.** | **525 nm refl.** | **630 nm refl.** | **645 nm refl.** | **660 nm refl.** | **940 nm refl.** | **970 nm refl.** | **Seed area (mm^2^)** | **Seed convex area (mm2)** | **Seed eccentricity** | **Seed length (mm)** | **Seed LWR** | **Seed perimeter (mm)** | **Seed roundness** | **Seed width (mm)** |
| --- | --- | --- | --- | --- | --- | --- | --- | --- | --- | --- | --- | --- | --- | --- | --- | --- |
| A404 | 13.443 | 40.546 | 55.727 | 73.152 | 82.427 | 86.385 | 171.965 | 172.135 | 17.918 | 18.246 | 0.841 | 6.533 | 1.872 | 17.315 | 0.751 | 3.507 |
| A412 | 14.173 | 41.809 | 57.594 | 76.183 | 89.457 | 95.267 | 176.700 | 176.187 | 17.419 | 17.683 | 0.828 | 6.311 | 1.797 | 16.839 | 0.772 | 3.521 |
| A422 | 14.144 | 41.234 | 56.854 | 78.154 | 89.438 | 94.339 | 176.685 | 176.245 | 16.458 | 16.720 | 0.874 | 6.583 | 2.075 | 16.879 | 0.724 | 3.186 |
| A438 | 14.106 | 40.926 | 57.649 | 79.630 | 92.017 | 97.366 | 179.150 | 178.593 | 14.623 | 14.888 | 0.835 | 5.846 | 1.834 | 15.505 | 0.764 | 3.199 |
| A1004 | 13.282 | 41.783 | 59.491 | 78.621 | 89.652 | 94.333 | 173.093 | 172.936 | 17.835 | 18.185 | 0.850 | 6.614 | 1.932 | 17.417 | 0.738 | 3.447 |
| A1005 | 15.179 | 45.181 | 60.753 | 79.334 | 90.993 | 96.130 | 178.046 | 177.715 | 15.850 | 16.124 | 0.834 | 6.066 | 1.821 | 16.120 | 0.766 | 3.334 |
| A1009 | 13.498 | 40.057 | 54.397 | 71.843 | 82.954 | 87.993 | 176.792 | 176.780 | 15.819 | 16.222 | 0.868 | 6.410 | 2.054 | 16.797 | 0.705 | 3.157 |
| A1010 | 14.323 | 42.474 | 57.889 | 77.460 | 88.726 | 93.680 | 176.111 | 175.720 | 17.695 | 18.011 | 0.860 | 6.670 | 1.976 | 17.361 | 0.737 | 3.389 |
| A1011 | 13.667 | 40.012 | 55.914 | 77.423 | 89.772 | 95.197 | 181.747 | 181.422 | 15.433 | 15.682 | 0.844 | 6.069 | 1.875 | 15.999 | 0.757 | 3.243 |
| A1012 | 14.224 | 44.168 | 61.053 | 79.921 | 92.558 | 97.868 | 178.501 | 178.266 | 18.626 | 18.946 | 0.829 | 6.548 | 1.812 | 17.529 | 0.762 | 3.636 |
| A1013 | 13.939 | 39.225 | 54.450 | 73.585 | 87.312 | 93.299 | 179.883 | 179.919 | 16.707 | 16.940 | 0.827 | 6.194 | 1.809 | 16.453 | 0.775 | 3.447 |
| A1014 | 15.186 | 48.104 | 64.181 | 80.229 | 91.361 | 96.149 | 175.748 | 175.834 | 16.041 | 16.412 | 0.880 | 6.574 | 2.117 | 16.800 | 0.713 | 3.116 |
| A1015 | 14.611 | 44.125 | 61.577 | 81.933 | 94.706 | 100.104 | 179.133 | 178.685 | 17.563 | 17.822 | 0.836 | 6.394 | 1.830 | 16.925 | 0.769 | 3.500 |
| A1018 | 14.297 | 40.519 | 56.878 | 78.739 | 89.596 | 94.116 | 174.477 | 174.584 | 17.127 | 17.438 | 0.854 | 6.489 | 1.935 | 17.352 | 0.728 | 3.370 |
| A1020 | 14.167 | 41.804 | 57.980 | 77.888 | 87.694 | 91.799 | 174.444 | 174.509 | 17.617 | 17.883 | 0.853 | 6.574 | 1.928 | 17.331 | 0.742 | 3.422 |
| A1021 | 14.397 | 44.373 | 59.325 | 77.259 | 86.806 | 90.878 | 175.404 | 175.749 | 12.298 | 12.574 | 0.883 | 5.804 | 2.151 | 14.838 | 0.701 | 2.711 |
| A1022 | 14.887 | 46.543 | 62.216 | 77.921 | 89.439 | 94.434 | 173.899 | 173.745 | 21.471 | 21.801 | 0.836 | 7.067 | 1.826 | 18.766 | 0.765 | 3.872 |
| A1023 | 13.723 | 43.135 | 58.819 | 77.346 | 88.885 | 93.969 | 178.602 | 178.353 | 18.134 | 18.467 | 0.829 | 6.444 | 1.802 | 17.306 | 0.759 | 3.591 |
| A1027 | 14.289 | 42.321 | 58.658 | 78.733 | 89.484 | 94.078 | 177.986 | 178.059 | 15.280 | 15.586 | 0.853 | 6.133 | 1.939 | 16.255 | 0.731 | 3.179 |
| A1028 | 14.610 | 44.498 | 61.500 | 82.292 | 93.796 | 98.629 | 179.715 | 179.544 | 16.314 | 16.602 | 0.837 | 6.198 | 1.852 | 16.444 | 0.758 | 3.363 |
| A1029 | 14.371 | 40.151 | 55.022 | 74.133 | 84.479 | 89.008 | 169.149 | 168.652 | 17.524 | 17.808 | 0.813 | 6.223 | 1.733 | 16.811 | 0.779 | 3.603 |
| A1031 | 13.956 | 41.644 | 57.852 | 78.865 | 90.528 | 95.406 | 178.636 | 178.631 | 17.011 | 17.274 | 0.810 | 6.093 | 1.719 | 16.474 | 0.786 | 3.560 |
| A1032 | 13.151 | 37.180 | 51.527 | 69.983 | 80.605 | 85.169 | 172.714 | 173.021 | 15.876 | 16.153 | 0.833 | 6.069 | 1.825 | 16.149 | 0.764 | 3.339 |
| A1033 | 14.040 | 42.814 | 58.590 | 76.671 | 88.588 | 93.832 | 175.496 | 175.177 | 15.855 | 16.233 | 0.860 | 6.368 | 2.005 | 16.826 | 0.705 | 3.197 |
| A1034 | 14.456 | 43.731 | 58.850 | 76.475 | 87.857 | 92.875 | 176.339 | 175.998 | 18.050 | 18.356 | 0.819 | 6.356 | 1.756 | 17.199 | 0.767 | 3.629 |
| A1036 | 13.978 | 41.814 | 58.853 | 77.751 | 91.658 | 97.610 | 179.341 | 178.970 | 17.313 | 17.584 | 0.831 | 6.319 | 1.808 | 17.033 | 0.755 | 3.501 |
| A1037 | 13.858 | 41.710 | 58.001 | 76.813 | 88.998 | 94.145 | 178.296 | 178.772 | 18.276 | 18.615 | 0.882 | 7.057 | 2.156 | 17.945 | 0.710 | 3.299 |
| A1038 | 13.703 | 39.980 | 54.734 | 69.973 | 81.133 | 85.998 | 170.409 | 170.833 | 17.957 | 18.323 | 0.872 | 6.883 | 2.084 | 17.855 | 0.710 | 3.335 |
| A1039 | 13.878 | 40.668 | 56.229 | 73.784 | 86.202 | 91.653 | 175.233 | 175.166 | 19.014 | 19.317 | 0.876 | 7.104 | 2.082 | 18.439 | 0.709 | 3.420 |
| A1040 | 16.089 | 45.255 | 60.958 | 79.947 | 89.819 | 93.997 | 173.578 | 173.662 | 15.764 | 16.004 | 0.855 | 6.227 | 1.935 | 16.198 | 0.754 | 3.226 |
| A1041 | 14.642 | 48.729 | 70.324 | 91.626 | 102.174 | 106.501 | 179.549 | 179.254 | 17.751 | 18.045 | 0.846 | 6.524 | 1.881 | 17.179 | 0.755 | 3.474 |
| A1042 | 13.171 | 39.958 | 56.075 | 74.993 | 87.431 | 92.732 | 179.828 | 180.041 | 16.589 | 16.869 | 0.858 | 6.435 | 1.966 | 16.804 | 0.737 | 3.290 |
| A1043 | 14.572 | 46.903 | 67.437 | 86.191 | 99.575 | 105.307 | 179.354 | 178.797 | 15.411 | 15.650 | 0.832 | 5.969 | 1.815 | 15.818 | 0.774 | 3.298 |
| A1044 | 13.294 | 40.891 | 56.628 | 75.202 | 87.860 | 93.320 | 175.473 | 175.191 | 18.276 | 18.541 | 0.809 | 6.313 | 1.713 | 17.056 | 0.789 | 3.695 |
| A1045 | 13.639 | 42.004 | 57.533 | 74.392 | 85.306 | 89.966 | 173.127 | 173.281 | 18.569 | 18.919 | 0.845 | 6.690 | 1.897 | 17.723 | 0.742 | 3.546 |
| A1047 | 13.131 | 37.002 | 53.067 | 74.768 | 87.611 | 93.242 | 177.820 | 177.762 | 15.041 | 15.299 | 0.843 | 5.992 | 1.878 | 15.815 | 0.755 | 3.205 |
| A1049 | 13.398 | 41.736 | 57.083 | 71.607 | 85.665 | 91.878 | 175.672 | 175.358 | 15.997 | 16.263 | 0.816 | 5.961 | 1.749 | 16.068 | 0.777 | 3.423 |
| A1050 | 14.492 | 43.314 | 59.212 | 76.322 | 88.105 | 93.124 | 173.841 | 173.697 | 17.670 | 17.931 | 0.828 | 6.354 | 1.797 | 16.897 | 0.776 | 3.546 |
| A1051 | 13.961 | 43.692 | 59.438 | 75.034 | 87.658 | 93.164 | 172.924 | 172.576 | 17.357 | 17.637 | 0.826 | 6.302 | 1.802 | 16.799 | 0.773 | 3.518 |
| A1052 | 12.966 | 38.956 | 55.445 | 75.904 | 89.566 | 95.508 | 180.204 | 179.981 | 17.119 | 17.375 | 0.853 | 6.480 | 1.933 | 16.898 | 0.751 | 3.366 |
| A1054 | 13.783 | 43.182 | 58.542 | 76.917 | 88.599 | 93.577 | 177.668 | 177.555 | 16.099 | 16.421 | 0.852 | 6.304 | 1.936 | 16.568 | 0.737 | 3.270 |
| A1056 | 15.160 | 48.053 | 69.994 | 94.813 | 104.884 | 109.110 | 182.187 | 181.636 | 14.878 | 15.163 | 0.874 | 6.264 | 2.072 | 16.137 | 0.717 | 3.036 |
| A1057 | 13.987 | 40.345 | 56.708 | 75.875 | 89.734 | 95.798 | 175.676 | 175.189 | 17.814 | 18.082 | 0.836 | 6.450 | 1.836 | 17.055 | 0.768 | 3.528 |
| A1058 | 14.362 | 43.377 | 58.832 | 75.747 | 90.286 | 96.602 | 180.850 | 180.714 | 18.022 | 18.269 | 0.830 | 6.436 | 1.806 | 17.061 | 0.778 | 3.572 |
| A1060 | 14.779 | 43.941 | 60.061 | 79.118 | 90.697 | 95.615 | 176.655 | 176.603 | 19.642 | 19.925 | 0.871 | 7.159 | 2.052 | 18.395 | 0.728 | 3.502 |
| A1061 | 13.729 | 44.341 | 61.296 | 81.192 | 94.594 | 100.375 | 181.183 | 180.872 | 17.731 | 17.997 | 0.838 | 6.453 | 1.844 | 17.077 | 0.763 | 3.508 |
| A1062 | 14.377 | 43.142 | 59.312 | 78.229 | 89.267 | 94.033 | 173.348 | 173.017 | 17.329 | 17.683 | 0.845 | 6.464 | 1.887 | 17.347 | 0.732 | 3.433 |
| A1063 | 13.942 | 41.744 | 57.569 | 77.026 | 88.891 | 94.200 | 178.394 | 178.114 | 17.547 | 17.900 | 0.874 | 6.804 | 2.070 | 17.730 | 0.706 | 3.295 |
| A1064 | 13.752 | 40.998 | 55.871 | 74.672 | 85.267 | 89.913 | 174.251 | 174.185 | 17.234 | 17.661 | 0.899 | 7.127 | 2.309 | 17.933 | 0.673 | 3.102 |
| A1065 | 13.529 | 41.589 | 58.062 | 76.765 | 89.985 | 95.725 | 178.955 | 178.726 | 17.493 | 17.837 | 0.864 | 6.672 | 2.004 | 17.466 | 0.723 | 3.344 |
| A1066 | 14.126 | 42.023 | 58.009 | 77.406 | 88.737 | 93.605 | 176.260 | 176.369 | 18.167 | 18.424 | 0.823 | 6.402 | 1.772 | 17.132 | 0.778 | 3.620 |
| A1067 | 15.180 | 45.310 | 60.633 | 80.204 | 90.785 | 95.449 | 177.645 | 177.424 | 19.319 | 19.648 | 0.855 | 6.929 | 1.950 | 18.125 | 0.739 | 3.570 |
| A1068 | 14.380 | 44.639 | 60.592 | 79.619 | 89.715 | 94.107 | 174.490 | 174.198 | 18.816 | 19.155 | 0.843 | 6.711 | 1.883 | 17.735 | 0.750 | 3.584 |
| A1073 | 13.770 | 41.944 | 59.964 | 80.026 | 90.670 | 95.389 | 173.842 | 173.166 | 16.523 | 16.818 | 0.856 | 6.419 | 1.965 | 16.757 | 0.738 | 3.290 |
| A1075 | 13.904 | 41.958 | 57.776 | 75.461 | 88.794 | 94.616 | 180.293 | 180.399 | 14.587 | 14.882 | 0.848 | 5.936 | 1.896 | 15.685 | 0.744 | 3.134 |
| A1076 | 14.199 | 47.909 | 68.699 | 90.345 | 101.357 | 105.894 | 177.600 | 177.267 | 17.117 | 17.436 | 0.880 | 6.806 | 2.133 | 17.433 | 0.707 | 3.210 |
| A1077 | 13.910 | 42.457 | 57.708 | 76.489 | 88.764 | 94.171 | 176.734 | 176.262 | 17.516 | 17.800 | 0.828 | 6.338 | 1.805 | 16.899 | 0.770 | 3.526 |
| A1078 | 13.269 | 41.213 | 57.439 | 76.000 | 89.513 | 95.353 | 178.656 | 178.406 | 18.291 | 18.589 | 0.848 | 6.675 | 1.911 | 17.533 | 0.748 | 3.504 |
| A1079 | 12.930 | 40.845 | 59.810 | 78.320 | 91.022 | 96.504 | 174.299 | 173.661 | 19.085 | 19.370 | 0.842 | 6.734 | 1.867 | 17.754 | 0.760 | 3.619 |
| A1080 | 13.456 | 39.220 | 55.330 | 76.492 | 88.848 | 94.154 | 180.897 | 180.999 | 18.024 | 18.315 | 0.860 | 6.729 | 1.971 | 17.489 | 0.740 | 3.421 |
| A1081 | 13.481 | 40.632 | 56.355 | 73.090 | 85.167 | 90.432 | 174.265 | 174.545 | 16.576 | 16.924 | 0.839 | 6.280 | 1.874 | 16.713 | 0.746 | 3.377 |
| A1082 | 14.734 | 46.233 | 62.552 | 82.959 | 94.631 | 99.657 | 179.340 | 178.776 | 17.986 | 18.248 | 0.795 | 6.160 | 1.660 | 16.793 | 0.801 | 3.721 |
| A1083 | 14.075 | 42.951 | 58.463 | 79.218 | 92.053 | 97.627 | 181.523 | 181.005 | 17.178 | 17.453 | 0.841 | 6.387 | 1.867 | 16.884 | 0.756 | 3.435 |
| A1084 | 14.329 | 40.986 | 57.143 | 78.235 | 89.060 | 93.736 | 176.525 | 176.732 | 17.424 | 17.684 | 0.823 | 6.273 | 1.777 | 16.801 | 0.775 | 3.546 |
| A1086 | 15.557 | 50.135 | 71.467 | 92.471 | 104.766 | 109.948 | 183.244 | 182.841 | 15.265 | 15.504 | 0.831 | 5.927 | 1.811 | 15.729 | 0.774 | 3.282 |
| A1088 | 14.164 | 46.724 | 64.614 | 85.739 | 98.197 | 103.542 | 181.456 | 180.894 | 18.132 | 18.400 | 0.845 | 6.603 | 1.895 | 17.477 | 0.750 | 3.496 |
| A1092 | 15.621 | 46.324 | 62.753 | 83.415 | 94.631 | 99.489 | 178.420 | 177.955 | 17.078 | 17.368 | 0.855 | 6.501 | 1.945 | 16.985 | 0.744 | 3.355 |
| A1093 | 13.975 | 42.123 | 57.750 | 74.859 | 87.480 | 92.905 | 175.773 | 175.785 | 13.467 | 13.764 | 0.842 | 5.675 | 1.887 | 15.064 | 0.744 | 3.031 |
| A1094 | 14.805 | 45.026 | 60.759 | 80.885 | 93.201 | 98.602 | 179.967 | 179.478 | 18.830 | 19.122 | 0.832 | 6.602 | 1.820 | 17.535 | 0.768 | 3.641 |
| A1095 | 14.055 | 43.531 | 60.841 | 82.717 | 93.408 | 97.939 | 176.801 | 176.367 | 18.308 | 18.633 | 0.843 | 6.623 | 1.881 | 17.550 | 0.746 | 3.540 |
| A1096 | 14.506 | 44.929 | 61.715 | 84.381 | 96.197 | 101.236 | 181.315 | 180.829 | 16.486 | 16.751 | 0.836 | 6.207 | 1.835 | 16.428 | 0.767 | 3.392 |
| A1097 | 13.451 | 42.329 | 58.252 | 79.607 | 91.227 | 96.316 | 178.953 | 178.560 | 16.927 | 17.243 | 0.873 | 6.680 | 2.069 | 17.262 | 0.713 | 3.240 |
| A1099 | 13.809 | 40.402 | 55.636 | 73.323 | 84.223 | 88.852 | 171.922 | 172.073 | 14.557 | 14.832 | 0.850 | 5.954 | 1.927 | 15.588 | 0.748 | 3.107 |
| A1102 | 14.281 | 40.872 | 57.234 | 76.847 | 87.297 | 91.671 | 175.192 | 175.359 | 16.442 | 16.726 | 0.874 | 6.582 | 2.067 | 16.937 | 0.720 | 3.193 |
| A1103 | 14.154 | 46.133 | 62.851 | 79.527 | 92.790 | 98.553 | 177.142 | 176.772 | 21.595 | 21.911 | 0.851 | 7.281 | 1.927 | 19.144 | 0.741 | 3.792 |
| A1104 | 15.802 | 49.484 | 66.069 | 87.010 | 98.015 | 102.693 | 179.128 | 178.450 | 18.175 | 18.486 | 0.857 | 6.726 | 1.963 | 17.607 | 0.735 | 3.446 |
| A1105 | 15.070 | 50.050 | 70.525 | 93.794 | 105.054 | 109.813 | 179.659 | 178.613 | 18.654 | 18.926 | 0.843 | 6.672 | 1.877 | 17.582 | 0.758 | 3.567 |
| A1106 | 12.255 | 34.076 | 49.245 | 66.344 | 78.687 | 84.137 | 175.211 | 175.687 | 12.258 | 12.537 | 0.891 | 5.909 | 2.243 | 14.895 | 0.694 | 2.656 |
| A1107 | 14.452 | 42.303 | 59.192 | 79.716 | 90.183 | 94.730 | 173.267 | 172.783 | 15.806 | 16.073 | 0.809 | 5.890 | 1.724 | 15.935 | 0.782 | 3.434 |
| A1109 | 14.134 | 43.459 | 60.332 | 83.885 | 96.179 | 101.430 | 181.720 | 181.014 | 16.935 | 17.200 | 0.852 | 6.447 | 1.927 | 16.865 | 0.748 | 3.358 |
| A1111 | 15.195 | 47.335 | 64.912 | 86.259 | 98.844 | 104.225 | 181.024 | 180.347 | 17.210 | 17.498 | 0.850 | 6.471 | 1.916 | 17.092 | 0.743 | 3.391 |
| A1112 | 14.525 | 45.501 | 62.119 | 82.293 | 95.852 | 101.810 | 183.285 | 182.718 | 19.251 | 19.560 | 0.853 | 6.888 | 1.938 | 18.020 | 0.745 | 3.569 |
| A1113 | 13.576 | 40.353 | 56.537 | 74.567 | 86.924 | 92.311 | 175.389 | 175.287 | 18.932 | 19.262 | 0.882 | 7.183 | 2.143 | 18.386 | 0.707 | 3.369 |
| A1114 | 13.868 | 41.387 | 56.970 | 73.235 | 87.993 | 94.607 | 178.007 | 177.586 | 16.917 | 17.246 | 0.881 | 6.780 | 2.129 | 17.334 | 0.707 | 3.193 |
| A1115 | 13.831 | 41.907 | 58.968 | 81.273 | 93.735 | 99.095 | 182.421 | 182.308 | 18.463 | 18.797 | 0.867 | 6.902 | 2.028 | 17.913 | 0.723 | 3.417 |
| A1116 | 13.745 | 42.535 | 59.556 | 79.172 | 91.592 | 96.840 | 178.891 | 178.986 | 17.561 | 17.974 | 0.878 | 6.865 | 2.105 | 17.719 | 0.704 | 3.271 |
| A1117 | 15.335 | 45.624 | 61.029 | 80.863 | 91.075 | 95.482 | 177.612 | 177.415 | 16.530 | 16.773 | 0.861 | 6.439 | 1.972 | 16.668 | 0.746 | 3.272 |
| A1118 | 15.794 | 46.604 | 62.620 | 80.739 | 93.265 | 98.732 | 178.229 | 177.628 | 17.242 | 17.486 | 0.851 | 6.480 | 1.913 | 16.878 | 0.759 | 3.395 |
| A1119 | 14.822 | 44.814 | 61.124 | 79.037 | 90.448 | 95.321 | 174.963 | 175.189 | 19.204 | 19.553 | 0.863 | 6.991 | 2.006 | 18.189 | 0.728 | 3.508 |
| A1120 | 14.662 | 45.736 | 61.791 | 80.901 | 92.198 | 97.052 | 177.599 | 177.149 | 18.447 | 18.751 | 0.867 | 6.887 | 2.019 | 17.826 | 0.732 | 3.419 |
| A1121 | 16.116 | 49.943 | 66.052 | 86.202 | 96.639 | 101.118 | 180.520 | 180.388 | 13.396 | 13.690 | 0.882 | 6.048 | 2.146 | 15.403 | 0.709 | 2.830 |
| A1122 | 14.405 | 45.250 | 60.642 | 78.240 | 90.552 | 95.980 | 178.055 | 177.742 | 16.148 | 16.482 | 0.867 | 6.446 | 2.027 | 16.836 | 0.717 | 3.197 |
| A1123 | 14.882 | 49.669 | 65.223 | 81.671 | 92.476 | 97.187 | 178.254 | 178.154 | 18.354 | 18.780 | 0.866 | 6.881 | 2.031 | 17.990 | 0.711 | 3.409 |
| A1124 | 14.785 | 47.733 | 63.601 | 82.347 | 93.033 | 97.690 | 178.005 | 177.842 | 17.637 | 18.072 | 0.880 | 6.921 | 2.145 | 17.869 | 0.693 | 3.260 |
| A1126 | 14.603 | 44.697 | 60.180 | 78.249 | 88.714 | 93.243 | 175.497 | 175.745 | 19.742 | 20.134 | 0.857 | 7.019 | 1.958 | 18.355 | 0.736 | 3.596 |
| A1127 | 14.632 | 41.561 | 56.980 | 72.160 | 85.967 | 92.110 | 173.760 | 173.607 | 15.803 | 16.082 | 0.860 | 6.335 | 1.998 | 16.478 | 0.732 | 3.194 |
| A1129 | 15.743 | 47.767 | 63.991 | 83.754 | 95.194 | 100.144 | 178.363 | 177.970 | 17.176 | 17.543 | 0.884 | 6.879 | 2.163 | 17.689 | 0.692 | 3.198 |
| A1130 | 14.790 | 44.014 | 59.612 | 80.609 | 92.812 | 98.191 | 181.326 | 181.156 | 15.863 | 16.195 | 0.847 | 6.197 | 1.895 | 16.459 | 0.735 | 3.283 |
| A1131 | 14.269 | 42.341 | 58.657 | 79.055 | 89.904 | 94.509 | 177.786 | 177.849 | 16.602 | 16.965 | 0.853 | 6.428 | 1.958 | 16.862 | 0.733 | 3.313 |
| A1132 | 14.513 | 47.291 | 63.581 | 82.285 | 93.300 | 98.130 | 177.793 | 177.834 | 17.441 | 17.828 | 0.876 | 6.822 | 2.097 | 17.682 | 0.705 | 3.267 |
| A1133 | 13.765 | 40.972 | 56.811 | 76.629 | 88.613 | 93.895 | 177.134 | 176.657 | 17.547 | 17.871 | 0.868 | 6.743 | 2.033 | 17.470 | 0.721 | 3.328 |
| A1134 | 13.269 | 41.039 | 56.760 | 76.087 | 87.784 | 92.973 | 175.403 | 174.864 | 16.350 | 16.674 | 0.849 | 6.325 | 1.920 | 16.635 | 0.741 | 3.312 |
| A1139 | 13.059 | 40.736 | 57.092 | 74.826 | 87.821 | 93.536 | 175.650 | 175.316 | 18.579 | 18.865 | 0.829 | 6.523 | 1.800 | 17.393 | 0.771 | 3.633 |
| A1140 | 15.452 | 46.445 | 62.144 | 81.643 | 93.244 | 98.349 | 179.195 | 178.642 | 16.554 | 16.824 | 0.823 | 6.136 | 1.788 | 16.448 | 0.769 | 3.453 |
| A1141 | 14.205 | 44.887 | 60.611 | 79.699 | 90.748 | 95.612 | 176.458 | 176.111 | 15.851 | 16.130 | 0.822 | 5.979 | 1.769 | 16.090 | 0.768 | 3.388 |
| A1142 | 14.047 | 39.830 | 53.988 | 70.385 | 80.731 | 85.363 | 168.336 | 168.136 | 17.525 | 17.872 | 0.905 | 7.292 | 2.379 | 18.125 | 0.670 | 3.078 |
| A1143 | 14.652 | 43.177 | 58.578 | 76.933 | 88.954 | 94.215 | 174.534 | 173.997 | 19.426 | 19.749 | 0.836 | 6.744 | 1.842 | 17.892 | 0.762 | 3.680 |
| A1144 | 14.271 | 40.649 | 54.529 | 71.470 | 80.433 | 84.417 | 170.339 | 170.639 | 14.096 | 14.648 | 0.881 | 6.268 | 2.183 | 16.279 | 0.669 | 2.911 |
| A1145 | 14.336 | 44.239 | 60.891 | 83.074 | 95.910 | 101.548 | 181.529 | 180.939 | 17.865 | 18.147 | 0.822 | 6.343 | 1.768 | 17.060 | 0.771 | 3.598 |
| A1146 | 15.130 | 44.074 | 58.737 | 78.089 | 90.511 | 95.984 | 178.585 | 178.206 | 16.342 | 16.642 | 0.843 | 6.266 | 1.881 | 16.577 | 0.748 | 3.343 |
| A1147 | 14.085 | 41.323 | 56.573 | 74.384 | 87.121 | 92.647 | 176.710 | 176.476 | 16.531 | 16.837 | 0.855 | 6.415 | 1.953 | 16.811 | 0.735 | 3.298 |
| A1148 | 13.966 | 40.842 | 53.994 | 70.686 | 80.967 | 85.568 | 171.538 | 171.437 | 16.375 | 16.682 | 0.882 | 6.707 | 2.159 | 17.094 | 0.702 | 3.122 |
| A1149 | 14.740 | 42.369 | 57.313 | 74.987 | 86.879 | 92.058 | 175.037 | 174.789 | 19.732 | 20.002 | 0.835 | 6.777 | 1.827 | 17.949 | 0.770 | 3.716 |
| A1150 | 14.716 | 45.719 | 61.094 | 78.576 | 89.188 | 93.806 | 176.974 | 177.349 | 15.925 | 16.173 | 0.836 | 6.101 | 1.840 | 16.360 | 0.755 | 3.331 |
| A1151 | 14.033 | 41.319 | 55.342 | 69.626 | 80.054 | 84.660 | 173.259 | 174.054 | 15.359 | 15.654 | 0.878 | 6.427 | 2.119 | 16.403 | 0.716 | 3.055 |
| A1152 | 13.493 | 39.979 | 55.562 | 72.710 | 85.305 | 90.823 | 175.476 | 175.873 | 18.328 | 18.629 | 0.828 | 6.480 | 1.797 | 17.300 | 0.770 | 3.617 |
| A1153 | 13.970 | 41.910 | 57.127 | 75.670 | 87.417 | 92.607 | 178.625 | 178.564 | 17.954 | 18.272 | 0.867 | 6.810 | 2.037 | 17.739 | 0.720 | 3.368 |
| A1154 | 15.405 | 45.401 | 59.680 | 76.442 | 87.228 | 92.024 | 175.088 | 174.818 | 18.345 | 18.620 | 0.845 | 6.666 | 1.901 | 17.480 | 0.755 | 3.527 |
| A1155 | 13.813 | 42.175 | 58.134 | 76.152 | 88.382 | 93.673 | 176.154 | 176.068 | 18.800 | 19.076 | 0.832 | 6.581 | 1.810 | 17.467 | 0.774 | 3.644 |
| A1156 | 14.949 | 42.546 | 56.748 | 73.775 | 85.311 | 90.450 | 175.184 | 175.021 | 15.751 | 16.047 | 0.862 | 6.357 | 2.028 | 16.464 | 0.729 | 3.172 |
| A1158 | 13.401 | 40.755 | 56.320 | 73.261 | 86.684 | 92.561 | 176.859 | 176.939 | 16.089 | 16.447 | 0.844 | 6.211 | 1.879 | 16.539 | 0.743 | 3.315 |
| A1159 | 14.160 | 43.934 | 61.189 | 82.761 | 95.034 | 100.271 | 178.713 | 178.024 | 18.401 | 18.697 | 0.836 | 6.561 | 1.834 | 17.443 | 0.759 | 3.586 |
| A1160 | 13.217 | 37.678 | 53.274 | 73.640 | 87.402 | 93.345 | 176.556 | 176.079 | 18.487 | 18.762 | 0.849 | 6.698 | 1.905 | 17.663 | 0.748 | 3.525 |
| A1161 | 13.037 | 40.699 | 57.324 | 77.301 | 89.335 | 94.474 | 176.247 | 176.249 | 18.390 | 18.669 | 0.832 | 6.526 | 1.824 | 17.319 | 0.770 | 3.594 |
| A1162 | 14.752 | 44.209 | 59.825 | 74.234 | 88.054 | 94.197 | 175.785 | 175.621 | 18.949 | 19.252 | 0.855 | 6.841 | 1.939 | 17.853 | 0.747 | 3.536 |
| A1163 | 14.508 | 45.622 | 61.470 | 79.209 | 90.500 | 95.330 | 175.978 | 175.758 | 17.093 | 17.424 | 0.870 | 6.686 | 2.052 | 17.174 | 0.729 | 3.271 |
| A1164 | 14.534 | 41.475 | 57.004 | 75.523 | 85.931 | 90.348 | 174.113 | 174.262 | 18.472 | 18.736 | 0.834 | 6.548 | 1.823 | 17.355 | 0.770 | 3.600 |
| A1167 | 14.140 | 45.048 | 61.355 | 80.389 | 91.475 | 96.266 | 176.374 | 175.952 | 20.978 | 21.385 | 0.853 | 7.198 | 1.943 | 18.937 | 0.733 | 3.727 |
| A1168 | 14.633 | 45.939 | 61.935 | 81.828 | 92.957 | 97.780 | 178.100 | 177.630 | 18.343 | 18.639 | 0.831 | 6.499 | 1.812 | 17.333 | 0.766 | 3.599 |
| A1171 | 15.209 | 45.344 | 61.554 | 83.197 | 95.410 | 100.696 | 181.582 | 181.096 | 19.761 | 20.105 | 0.842 | 6.869 | 1.874 | 18.144 | 0.755 | 3.677 |
| A1173 | 14.947 | 44.916 | 59.645 | 78.147 | 87.929 | 92.164 | 173.131 | 172.610 | 17.397 | 17.685 | 0.835 | 6.372 | 1.838 | 16.916 | 0.764 | 3.484 |
| A1174 | 13.333 | 42.379 | 58.262 | 77.176 | 90.427 | 96.245 | 180.111 | 179.462 | 17.251 | 17.532 | 0.811 | 6.165 | 1.732 | 16.735 | 0.774 | 3.578 |
| A1175 | 13.472 | 40.164 | 56.010 | 73.421 | 86.642 | 92.411 | 174.833 | 174.650 | 18.012 | 18.277 | 0.832 | 6.442 | 1.809 | 17.094 | 0.774 | 3.567 |
| A1176 | 14.900 | 45.622 | 60.870 | 79.416 | 91.797 | 97.245 | 180.830 | 180.973 | 16.718 | 17.083 | 0.847 | 6.377 | 1.906 | 17.029 | 0.727 | 3.363 |
| A1177 | 13.410 | 38.883 | 53.678 | 72.657 | 83.606 | 88.425 | 175.424 | 175.724 | 14.053 | 14.388 | 0.805 | 5.552 | 1.728 | 15.242 | 0.762 | 3.245 |
| A1178 | 14.995 | 44.526 | 63.406 | 83.763 | 95.022 | 99.780 | 178.583 | 178.942 | 13.646 | 13.992 | 0.890 | 6.254 | 2.256 | 15.924 | 0.677 | 2.802 |
| A1179 | 14.156 | 43.804 | 59.462 | 78.869 | 89.756 | 94.463 | 175.954 | 175.621 | 15.608 | 15.961 | 0.860 | 6.285 | 1.985 | 16.512 | 0.719 | 3.188 |
| A1180 | 14.683 | 43.607 | 59.537 | 75.811 | 89.378 | 95.384 | 175.691 | 175.621 | 15.740 | 16.029 | 0.825 | 5.985 | 1.789 | 16.122 | 0.761 | 3.362 |
| A1181 | 14.731 | 45.430 | 61.463 | 81.583 | 92.080 | 96.759 | 179.159 | 179.193 | 15.543 | 16.033 | 0.897 | 6.791 | 2.343 | 17.460 | 0.645 | 2.949 |
| A1182 | 14.642 | 46.425 | 62.437 | 82.456 | 94.636 | 99.969 | 180.315 | 179.869 | 16.615 | 16.887 | 0.829 | 6.169 | 1.798 | 16.487 | 0.767 | 3.439 |
| A1183 | 13.846 | 39.763 | 55.724 | 74.570 | 86.621 | 91.814 | 174.448 | 174.561 | 15.288 | 15.533 | 0.842 | 6.032 | 1.874 | 15.887 | 0.760 | 3.236 |
| A1185 | 14.466 | 42.528 | 57.795 | 77.409 | 88.082 | 92.617 | 176.170 | 175.757 | 15.014 | 15.264 | 0.847 | 6.028 | 1.902 | 15.797 | 0.755 | 3.182 |
| A1186 | 13.787 | 39.362 | 54.794 | 75.090 | 86.847 | 91.982 | 176.259 | 175.841 | 15.097 | 15.353 | 0.862 | 6.176 | 1.985 | 15.980 | 0.742 | 3.121 |
| A1187 | 14.017 | 43.465 | 59.947 | 80.906 | 92.919 | 98.133 | 181.368 | 181.110 | 16.205 | 16.555 | 0.858 | 6.384 | 1.978 | 16.838 | 0.720 | 3.245 |
| A1188 | 13.050 | 37.981 | 53.659 | 71.257 | 84.275 | 89.999 | 176.091 | 176.203 | 14.469 | 14.707 | 0.806 | 5.609 | 1.710 | 15.204 | 0.785 | 3.294 |
| A1189 | 13.966 | 40.542 | 55.793 | 74.733 | 86.728 | 92.023 | 173.974 | 173.451 | 15.967 | 16.217 | 0.814 | 5.936 | 1.732 | 15.953 | 0.788 | 3.435 |
| A1190 | 13.131 | 37.624 | 52.948 | 70.620 | 83.313 | 88.888 | 177.627 | 178.402 | 14.432 | 14.704 | 0.855 | 5.987 | 1.952 | 15.700 | 0.736 | 3.079 |
| A1191 | 13.030 | 41.400 | 56.846 | 71.205 | 84.970 | 91.084 | 176.034 | 175.900 | 15.288 | 15.603 | 0.845 | 6.058 | 1.884 | 16.054 | 0.744 | 3.223 |
| A1192 | 13.700 | 41.666 | 56.918 | 72.245 | 84.340 | 89.555 | 171.035 | 171.034 | 18.023 | 18.329 | 0.867 | 6.828 | 2.038 | 17.619 | 0.728 | 3.372 |
| A1193 | 13.934 | 39.781 | 53.761 | 71.287 | 82.213 | 87.057 | 174.650 | 174.803 | 14.621 | 14.916 | 0.842 | 5.897 | 1.872 | 15.627 | 0.751 | 3.165 |
| A1195 | 13.894 | 39.297 | 55.307 | 75.758 | 88.659 | 94.155 | 179.333 | 179.307 | 15.816 | 16.068 | 0.841 | 6.131 | 1.874 | 16.160 | 0.760 | 3.292 |
| A1196 | 13.724 | 36.676 | 49.008 | 64.669 | 73.771 | 77.839 | 169.426 | 169.801 | 13.565 | 13.824 | 0.852 | 5.769 | 1.929 | 15.082 | 0.749 | 3.003 |
| A1197 | 14.385 | 44.375 | 59.860 | 77.676 | 88.517 | 93.194 | 175.410 | 175.130 | 16.718 | 16.993 | 0.832 | 6.239 | 1.832 | 16.575 | 0.764 | 3.425 |
| A1199 | 13.385 | 41.187 | 57.249 | 76.063 | 87.898 | 92.970 | 177.988 | 177.992 | 17.513 | 17.833 | 0.843 | 6.466 | 1.882 | 17.090 | 0.752 | 3.457 |
| A1200 | 13.484 | 44.901 | 64.218 | 82.832 | 96.547 | 102.487 | 179.613 | 179.196 | 16.818 | 17.064 | 0.802 | 6.008 | 1.690 | 16.295 | 0.795 | 3.572 |
| A1201 | 14.957 | 43.387 | 58.239 | 78.015 | 89.873 | 95.030 | 179.100 | 178.592 | 16.496 | 16.751 | 0.793 | 5.884 | 1.651 | 16.103 | 0.798 | 3.574 |
| A1202 | 13.472 | 38.378 | 53.716 | 73.987 | 85.040 | 89.775 | 175.696 | 175.775 | 17.442 | 17.710 | 0.838 | 6.393 | 1.848 | 16.928 | 0.762 | 3.476 |
| A1203 | 13.725 | 40.368 | 54.708 | 69.604 | 79.749 | 84.138 | 168.119 | 168.355 | 17.476 | 17.821 | 0.854 | 6.579 | 1.937 | 17.433 | 0.725 | 3.405 |
| A1204 | 13.048 | 39.026 | 53.826 | 70.449 | 84.061 | 90.135 | 177.221 | 176.978 | 17.663 | 17.925 | 0.796 | 6.117 | 1.664 | 16.702 | 0.795 | 3.688 |
| A1206 | 14.392 | 41.562 | 57.691 | 78.354 | 89.192 | 93.810 | 176.421 | 176.454 | 13.225 | 13.557 | 0.871 | 5.897 | 2.060 | 15.311 | 0.710 | 2.877 |
| A1207 | 13.580 | 41.197 | 57.474 | 75.962 | 88.054 | 93.280 | 176.568 | 176.539 | 16.107 | 16.423 | 0.881 | 6.621 | 2.140 | 16.966 | 0.702 | 3.111 |
| A1208 | 14.140 | 39.910 | 54.031 | 70.805 | 80.976 | 85.369 | 172.703 | 173.148 | 16.261 | 16.550 | 0.831 | 6.137 | 1.823 | 16.340 | 0.765 | 3.384 |
| A1210 | 13.661 | 41.883 | 58.098 | 75.726 | 89.023 | 94.852 | 177.362 | 177.085 | 17.158 | 17.446 | 0.874 | 6.730 | 2.079 | 17.247 | 0.723 | 3.253 |
| A1211 | 14.039 | 42.859 | 58.744 | 77.794 | 89.005 | 93.806 | 177.502 | 177.581 | 16.084 | 16.355 | 0.791 | 5.820 | 1.652 | 15.948 | 0.795 | 3.532 |
| A1212 | 14.559 | 42.535 | 57.233 | 76.076 | 88.254 | 93.586 | 179.311 | 178.842 | 16.660 | 16.937 | 0.833 | 6.211 | 1.815 | 16.562 | 0.763 | 3.428 |
| A1213 | 14.538 | 42.830 | 58.998 | 77.182 | 88.518 | 93.315 | 173.010 | 173.074 | 18.799 | 19.109 | 0.829 | 6.586 | 1.813 | 17.568 | 0.765 | 3.650 |
| A1214 | 14.345 | 41.161 | 57.132 | 77.810 | 89.993 | 95.436 | 178.012 | 177.548 | 17.177 | 17.437 | 0.819 | 6.197 | 1.758 | 16.640 | 0.779 | 3.539 |
| A1215 | 13.089 | 41.879 | 64.331 | 86.314 | 98.256 | 103.353 | 177.261 | 176.543 | 17.560 | 17.815 | 0.816 | 6.241 | 1.746 | 16.777 | 0.783 | 3.589 |
| A1216 | 13.697 | 40.144 | 56.434 | 78.688 | 89.628 | 94.369 | 174.913 | 174.677 | 16.090 | 16.347 | 0.837 | 6.143 | 1.842 | 16.282 | 0.762 | 3.345 |
| A1217 | 13.573 | 39.853 | 55.821 | 75.646 | 88.303 | 93.710 | 176.686 | 176.592 | 16.619 | 16.875 | 0.835 | 6.218 | 1.833 | 16.493 | 0.766 | 3.407 |
| A1218 | 14.475 | 43.090 | 59.997 | 82.800 | 94.849 | 99.875 | 181.699 | 181.673 | 19.055 | 19.333 | 0.855 | 6.852 | 1.938 | 17.847 | 0.750 | 3.545 |
| A1219 | 14.373 | 43.137 | 58.984 | 75.689 | 88.136 | 93.484 | 175.460 | 175.635 | 18.202 | 18.493 | 0.844 | 6.590 | 1.875 | 17.361 | 0.758 | 3.525 |
| A1220 | 14.738 | 43.595 | 59.049 | 77.747 | 88.852 | 93.687 | 174.841 | 174.442 | 17.884 | 18.190 | 0.853 | 6.635 | 1.931 | 17.566 | 0.734 | 3.451 |
| A1221 | 14.563 | 47.560 | 63.537 | 81.247 | 91.396 | 95.809 | 177.202 | 177.046 | 17.482 | 17.835 | 0.869 | 6.778 | 2.089 | 17.506 | 0.715 | 3.295 |
| A1223 | 14.472 | 43.230 | 58.346 | 71.969 | 85.592 | 91.745 | 174.860 | 174.740 | 17.697 | 18.029 | 0.874 | 6.842 | 2.081 | 17.609 | 0.717 | 3.306 |
| A1225 | 13.295 | 40.609 | 55.298 | 71.254 | 82.956 | 88.068 | 173.032 | 172.966 | 15.382 | 15.630 | 0.837 | 6.010 | 1.844 | 15.906 | 0.763 | 3.267 |
| A1226 | 13.045 | 43.391 | 63.943 | 82.662 | 95.433 | 100.936 | 177.126 | 176.691 | 17.916 | 18.222 | 0.861 | 6.731 | 1.984 | 17.509 | 0.735 | 3.403 |
| A1227 | 14.704 | 46.386 | 63.698 | 84.010 | 96.917 | 102.532 | 180.846 | 180.134 | 19.123 | 19.444 | 0.863 | 6.974 | 2.007 | 18.083 | 0.733 | 3.500 |
| A1228 | 14.183 | 42.475 | 58.477 | 78.670 | 90.725 | 95.936 | 177.031 | 176.238 | 17.552 | 17.830 | 0.838 | 6.417 | 1.843 | 16.986 | 0.764 | 3.487 |
| A1229 | 15.820 | 44.576 | 60.611 | 80.354 | 90.814 | 95.327 | 177.350 | 177.398 | 18.564 | 18.903 | 0.861 | 6.863 | 1.995 | 17.856 | 0.731 | 3.459 |
| A1230 | 14.575 | 49.375 | 67.976 | 88.515 | 100.267 | 105.353 | 180.452 | 179.574 | 16.911 | 17.263 | 0.861 | 6.534 | 1.987 | 17.066 | 0.729 | 3.306 |
| A1231 | 14.957 | 42.677 | 59.126 | 79.863 | 91.870 | 97.086 | 176.038 | 175.620 | 15.040 | 15.328 | 0.872 | 6.281 | 2.061 | 16.279 | 0.716 | 3.059 |
| A1232 | 14.264 | 41.867 | 57.193 | 78.197 | 90.519 | 95.889 | 179.896 | 179.553 | 15.756 | 16.056 | 0.821 | 5.956 | 1.770 | 16.051 | 0.768 | 3.379 |
| A1233 | 15.230 | 44.868 | 61.514 | 81.547 | 93.869 | 99.074 | 178.986 | 178.802 | 17.070 | 17.327 | 0.825 | 6.231 | 1.787 | 16.779 | 0.768 | 3.497 |
| A1234 | 13.990 | 41.328 | 57.484 | 78.526 | 89.675 | 94.454 | 177.088 | 177.070 | 19.368 | 19.712 | 0.858 | 6.959 | 1.966 | 18.351 | 0.726 | 3.551 |
| A1236 | 14.282 | 38.906 | 53.371 | 71.541 | 82.010 | 86.603 | 171.572 | 171.578 | 17.844 | 18.159 | 0.800 | 6.198 | 1.689 | 17.007 | 0.780 | 3.688 |
| A1237 | 14.041 | 42.316 | 59.691 | 80.870 | 91.898 | 96.544 | 178.210 | 178.110 | 17.364 | 17.636 | 0.827 | 6.303 | 1.799 | 16.826 | 0.770 | 3.519 |
| A1240 | 14.451 | 44.434 | 60.997 | 81.222 | 93.068 | 98.277 | 180.004 | 179.609 | 17.778 | 18.088 | 0.824 | 6.349 | 1.783 | 17.102 | 0.763 | 3.577 |
| A1242 | 14.458 | 46.455 | 62.997 | 83.675 | 95.123 | 100.061 | 180.501 | 180.209 | 16.845 | 17.133 | 0.836 | 6.276 | 1.835 | 16.867 | 0.748 | 3.428 |
| A1243 | 13.164 | 40.202 | 55.517 | 70.899 | 84.939 | 91.199 | 175.153 | 174.795 | 16.534 | 16.814 | 0.860 | 6.439 | 1.970 | 16.768 | 0.739 | 3.278 |
| A1244 | 14.899 | 45.862 | 62.604 | 83.583 | 94.955 | 99.899 | 181.065 | 180.634 | 16.378 | 16.668 | 0.888 | 6.747 | 2.184 | 17.124 | 0.701 | 3.097 |
| A1245 | 14.110 | 41.317 | 57.476 | 78.020 | 90.743 | 96.448 | 179.738 | 179.638 | 15.677 | 15.934 | 0.862 | 6.296 | 1.987 | 16.307 | 0.740 | 3.179 |
| A1246 | 12.417 | 35.948 | 51.466 | 70.170 | 83.832 | 89.907 | 175.836 | 175.475 | 14.744 | 15.009 | 0.851 | 6.014 | 1.926 | 15.776 | 0.744 | 3.137 |
| A1247 | 13.990 | 45.741 | 62.437 | 79.134 | 90.315 | 95.081 | 176.355 | 176.599 | 20.564 | 20.916 | 0.858 | 7.172 | 1.970 | 18.770 | 0.732 | 3.660 |
| A1248 | 13.729 | 40.987 | 56.324 | 76.031 | 88.245 | 93.608 | 177.326 | 176.939 | 16.168 | 16.451 | 0.848 | 6.260 | 1.907 | 16.484 | 0.747 | 3.297 |
| A1249 | 14.550 | 45.162 | 61.134 | 78.602 | 92.217 | 98.108 | 178.436 | 177.940 | 16.073 | 16.337 | 0.812 | 5.941 | 1.726 | 16.034 | 0.785 | 3.452 |
| A1250 | 14.356 | 41.137 | 55.237 | 70.909 | 82.635 | 87.702 | 173.055 | 172.972 | 16.009 | 16.299 | 0.858 | 6.314 | 1.963 | 16.454 | 0.741 | 3.229 |
| A1251 | 14.041 | 42.801 | 59.620 | 82.634 | 95.239 | 100.758 | 181.384 | 180.560 | 16.735 | 16.988 | 0.840 | 6.296 | 1.860 | 16.620 | 0.761 | 3.394 |
| A1253 | 14.071 | 44.208 | 61.850 | 83.849 | 97.606 | 103.563 | 183.724 | 182.991 | 17.399 | 17.690 | 0.844 | 6.455 | 1.885 | 17.018 | 0.754 | 3.441 |
| A1254 | 14.809 | 47.677 | 68.596 | 89.448 | 97.714 | 101.185 | 174.509 | 174.239 | 15.894 | 16.175 | 0.871 | 6.435 | 2.046 | 16.625 | 0.722 | 3.153 |
| A1255 | 15.005 | 50.160 | 70.504 | 91.515 | 102.391 | 107.059 | 180.262 | 179.582 | 16.776 | 17.071 | 0.836 | 6.273 | 1.841 | 16.668 | 0.759 | 3.420 |
| A1256 | 14.136 | 43.106 | 62.370 | 84.686 | 94.849 | 99.044 | 174.454 | 174.326 | 17.045 | 17.332 | 0.851 | 6.459 | 1.925 | 16.904 | 0.749 | 3.369 |
| A1257 | 14.288 | 44.037 | 61.136 | 83.318 | 95.312 | 100.424 | 181.796 | 181.653 | 20.228 | 20.508 | 0.839 | 6.908 | 1.853 | 18.220 | 0.766 | 3.738 |
| A1258 | 14.368 | 49.881 | 70.420 | 88.808 | 100.119 | 104.885 | 178.230 | 177.684 | 18.228 | 18.546 | 0.853 | 6.697 | 1.934 | 17.578 | 0.741 | 3.477 |
| A1260 | 14.414 | 41.757 | 57.957 | 77.465 | 90.894 | 96.681 | 178.607 | 178.377 | 16.235 | 16.517 | 0.847 | 6.285 | 1.912 | 16.531 | 0.747 | 3.310 |
| A1261 | 13.913 | 41.313 | 57.032 | 75.385 | 87.485 | 92.717 | 174.926 | 174.732 | 17.328 | 17.604 | 0.834 | 6.352 | 1.832 | 16.861 | 0.765 | 3.485 |
| A1262 | 13.993 | 40.660 | 56.412 | 77.740 | 90.510 | 96.052 | 179.763 | 179.296 | 16.896 | 17.241 | 0.869 | 6.629 | 2.041 | 17.264 | 0.713 | 3.262 |
| A1264 | 14.122 | 43.504 | 63.021 | 87.021 | 98.873 | 103.874 | 180.186 | 179.562 | 15.009 | 15.242 | 0.850 | 6.042 | 1.916 | 15.807 | 0.753 | 3.169 |
| A1265 | 14.355 | 43.090 | 58.509 | 74.672 | 89.932 | 96.736 | 179.569 | 179.267 | 16.727 | 16.985 | 0.849 | 6.363 | 1.901 | 16.868 | 0.744 | 3.354 |
| A1266 | 15.009 | 46.413 | 62.101 | 79.231 | 90.615 | 95.507 | 175.920 | 175.726 | 18.250 | 18.542 | 0.841 | 6.583 | 1.867 | 17.415 | 0.756 | 3.539 |
| A1268 | 12.423 | 35.989 | 50.699 | 67.854 | 78.528 | 83.121 | 170.263 | 170.378 | 16.723 | 17.022 | 0.860 | 6.492 | 1.982 | 16.921 | 0.733 | 3.288 |
| A1269 | 14.276 | 42.218 | 57.803 | 78.868 | 91.139 | 96.485 | 181.196 | 180.929 | 15.694 | 15.969 | 0.847 | 6.148 | 1.890 | 16.239 | 0.748 | 3.259 |
| A1270 | 13.892 | 41.726 | 58.556 | 77.613 | 88.713 | 93.465 | 175.016 | 175.011 | 14.504 | 14.787 | 0.868 | 6.141 | 2.048 | 15.873 | 0.723 | 3.018 |
| A1271 | 13.652 | 41.066 | 56.429 | 73.790 | 85.309 | 90.322 | 174.903 | 175.079 | 17.155 | 17.614 | 0.895 | 7.074 | 2.311 | 18.036 | 0.665 | 3.110 |
| A1272 | 14.736 | 45.915 | 62.782 | 83.295 | 94.279 | 98.882 | 179.471 | 179.579 | 16.553 | 16.821 | 0.853 | 6.375 | 1.929 | 16.674 | 0.748 | 3.312 |
| A1275 | 14.606 | 44.387 | 59.094 | 76.273 | 86.924 | 91.677 | 173.496 | 173.081 | 18.945 | 19.306 | 0.875 | 7.094 | 2.081 | 18.367 | 0.709 | 3.417 |
| A1276 | 13.092 | 38.817 | 52.926 | 68.120 | 78.797 | 83.499 | 172.883 | 173.578 | 15.652 | 15.987 | 0.878 | 6.513 | 2.140 | 16.744 | 0.701 | 3.078 |
| A1279 | 13.884 | 42.523 | 57.929 | 78.414 | 88.996 | 93.572 | 176.386 | 176.178 | 19.259 | 19.624 | 0.861 | 6.977 | 1.988 | 18.252 | 0.725 | 3.527 |
| A1282 | 14.578 | 46.959 | 64.983 | 87.144 | 99.563 | 104.883 | 182.535 | 181.853 | 17.109 | 17.449 | 0.864 | 6.607 | 2.000 | 17.222 | 0.724 | 3.316 |
| A1283 | 13.772 | 41.462 | 57.645 | 79.327 | 90.139 | 94.710 | 178.256 | 178.012 | 16.179 | 16.475 | 0.844 | 6.218 | 1.879 | 16.413 | 0.754 | 3.322 |
| A1285 | 13.153 | 40.977 | 57.534 | 80.021 | 92.942 | 98.579 | 181.447 | 181.101 | 16.061 | 16.330 | 0.808 | 5.932 | 1.726 | 16.077 | 0.780 | 3.459 |
| A1286 | 13.602 | 42.091 | 57.897 | 75.467 | 88.470 | 94.145 | 177.165 | 177.087 | 17.020 | 17.329 | 0.847 | 6.437 | 1.915 | 16.958 | 0.742 | 3.383 |
| A1287 | 14.707 | 42.276 | 57.898 | 77.351 | 89.476 | 94.815 | 178.738 | 178.584 | 16.859 | 17.146 | 0.857 | 6.475 | 1.955 | 16.909 | 0.741 | 3.321 |
| A1288 | 15.931 | 47.084 | 62.203 | 80.776 | 92.718 | 97.979 | 179.433 | 179.171 | 15.476 | 15.809 | 0.848 | 6.132 | 1.908 | 16.194 | 0.742 | 3.226 |
| A1289 | 12.882 | 38.223 | 54.419 | 72.545 | 88.242 | 95.251 | 179.556 | 179.322 | 17.438 | 17.698 | 0.851 | 6.509 | 1.910 | 17.027 | 0.755 | 3.413 |
| A1290 | 14.149 | 41.729 | 57.403 | 76.081 | 89.248 | 95.018 | 177.573 | 177.327 | 17.026 | 17.283 | 0.834 | 6.297 | 1.831 | 16.698 | 0.767 | 3.452 |
| A1291 | 14.654 | 44.174 | 59.586 | 80.915 | 92.947 | 98.289 | 178.280 | 177.528 | 16.975 | 17.215 | 0.805 | 6.055 | 1.695 | 16.394 | 0.793 | 3.581 |
| A1292 | 14.087 | 43.572 | 58.807 | 77.494 | 88.704 | 93.631 | 175.830 | 175.512 | 14.627 | 14.941 | 0.855 | 6.062 | 2.003 | 15.839 | 0.729 | 3.089 |
| A1293 | 14.664 | 42.792 | 57.222 | 74.974 | 85.050 | 89.469 | 173.114 | 172.961 | 13.960 | 14.248 | 0.868 | 6.018 | 2.038 | 15.611 | 0.719 | 2.967 |
| A1294 | 15.375 | 49.496 | 66.412 | 87.175 | 99.455 | 104.821 | 181.525 | 180.993 | 18.929 | 19.246 | 0.830 | 6.626 | 1.824 | 17.808 | 0.755 | 3.652 |
| A1295 | 13.802 | 41.276 | 57.958 | 77.802 | 90.566 | 96.014 | 175.922 | 175.667 | 16.242 | 16.514 | 0.865 | 6.443 | 2.010 | 16.654 | 0.736 | 3.220 |
| A1296 | 13.853 | 39.837 | 54.798 | 75.696 | 89.243 | 95.171 | 179.694 | 179.190 | 15.603 | 15.889 | 0.831 | 6.021 | 1.828 | 16.049 | 0.760 | 3.313 |
| A1297 | 13.561 | 41.563 | 57.076 | 74.173 | 86.731 | 92.110 | 174.892 | 174.928 | 18.100 | 18.507 | 0.876 | 6.961 | 2.096 | 17.905 | 0.709 | 3.336 |
| A1298 | 13.992 | 40.337 | 56.668 | 75.902 | 87.642 | 92.646 | 174.378 | 174.442 | 19.828 | 20.135 | 0.853 | 6.986 | 1.939 | 18.214 | 0.750 | 3.625 |
| A1300 | 14.019 | 46.299 | 66.266 | 87.195 | 99.033 | 104.026 | 178.859 | 178.495 | 18.109 | 18.434 | 0.843 | 6.594 | 1.884 | 17.487 | 0.745 | 3.513 |
| A1301 | 12.846 | 39.876 | 55.306 | 73.429 | 85.219 | 90.314 | 176.072 | 176.403 | 16.282 | 16.634 | 0.848 | 6.288 | 1.907 | 16.623 | 0.740 | 3.314 |
| A1302 | 14.182 | 45.137 | 60.384 | 77.160 | 88.065 | 92.756 | 174.387 | 174.368 | 18.853 | 19.257 | 0.877 | 7.109 | 2.103 | 18.399 | 0.703 | 3.393 |
| A1304 | 13.917 | 42.791 | 60.296 | 82.548 | 94.254 | 99.324 | 180.323 | 179.789 | 17.907 | 18.210 | 0.860 | 6.710 | 1.977 | 17.455 | 0.738 | 3.407 |
| A1305 | 14.338 | 44.781 | 60.478 | 78.719 | 90.088 | 95.029 | 174.828 | 174.465 | 18.704 | 19.062 | 0.879 | 7.155 | 2.157 | 18.493 | 0.692 | 3.343 |
| A1307 | 14.206 | 45.535 | 65.970 | 88.349 | 99.517 | 104.270 | 176.904 | 176.259 | 18.394 | 18.818 | 0.865 | 6.877 | 2.019 | 18.072 | 0.711 | 3.428 |
| A1308 | 13.587 | 43.869 | 60.277 | 78.816 | 90.153 | 94.987 | 175.336 | 174.976 | 19.027 | 19.361 | 0.857 | 6.882 | 1.953 | 18.031 | 0.735 | 3.533 |
| A1309 | 14.098 | 43.692 | 60.789 | 81.210 | 94.735 | 100.688 | 180.785 | 180.271 | 18.868 | 19.202 | 0.865 | 6.953 | 2.014 | 18.070 | 0.726 | 3.468 |
| A1310 | 14.156 | 40.522 | 55.095 | 74.374 | 85.607 | 90.547 | 176.069 | 176.309 | 16.086 | 16.376 | 0.844 | 6.200 | 1.878 | 16.358 | 0.754 | 3.311 |
| A1311 | 12.851 | 40.323 | 55.996 | 72.844 | 86.161 | 92.074 | 176.147 | 176.001 | 18.387 | 18.684 | 0.859 | 6.783 | 1.969 | 17.642 | 0.741 | 3.456 |
| A1312 | 14.538 | 45.164 | 60.585 | 75.938 | 88.545 | 94.000 | 175.918 | 175.846 | 18.626 | 18.927 | 0.872 | 6.986 | 2.061 | 18.007 | 0.722 | 3.404 |
| A1313 | 14.344 | 45.104 | 63.112 | 84.251 | 94.054 | 98.286 | 175.321 | 174.753 | 16.601 | 16.902 | 0.861 | 6.487 | 1.991 | 16.889 | 0.731 | 3.270 |
| A1314 | 16.749 | 51.199 | 67.769 | 88.271 | 98.557 | 102.990 | 178.888 | 178.453 | 15.492 | 15.779 | 0.866 | 6.314 | 2.028 | 16.369 | 0.726 | 3.135 |
| A1315 | 14.053 | 41.392 | 56.350 | 75.073 | 86.561 | 91.636 | 173.702 | 173.383 | 17.554 | 17.822 | 0.836 | 6.397 | 1.831 | 16.971 | 0.766 | 3.500 |
| A1316 | 13.931 | 46.770 | 66.975 | 88.859 | 99.644 | 104.107 | 180.140 | 179.797 | 17.436 | 17.717 | 0.807 | 6.145 | 1.706 | 16.688 | 0.785 | 3.618 |
| A1318 | 14.458 | 41.646 | 55.828 | 74.389 | 84.902 | 89.448 | 175.651 | 175.470 | 15.889 | 16.185 | 0.787 | 5.770 | 1.652 | 15.930 | 0.787 | 3.520 |
| A1319 | 14.478 | 42.530 | 58.392 | 79.362 | 90.405 | 95.194 | 179.349 | 179.436 | 17.863 | 18.117 | 0.816 | 6.279 | 1.735 | 16.877 | 0.787 | 3.624 |
| A1320 | 14.689 | 48.007 | 67.489 | 89.475 | 100.629 | 105.380 | 179.483 | 178.840 | 18.028 | 18.305 | 0.815 | 6.315 | 1.737 | 17.292 | 0.769 | 3.642 |
| A1321 | 13.359 | 39.931 | 55.676 | 76.615 | 88.960 | 94.333 | 178.135 | 177.727 | 17.502 | 17.764 | 0.829 | 6.349 | 1.817 | 16.879 | 0.770 | 3.516 |
| A1322 | 14.588 | 45.581 | 62.024 | 83.255 | 94.593 | 99.543 | 181.841 | 181.497 | 17.523 | 17.843 | 0.845 | 6.477 | 1.880 | 17.160 | 0.747 | 3.451 |
| A1323 | 13.997 | 41.379 | 57.241 | 77.387 | 88.699 | 93.535 | 178.216 | 178.375 | 16.507 | 16.802 | 0.848 | 6.325 | 1.902 | 16.652 | 0.748 | 3.335 |
| A1324 | 14.149 | 45.871 | 65.521 | 86.990 | 99.847 | 105.422 | 180.859 | 180.086 | 19.502 | 19.817 | 0.861 | 7.014 | 1.984 | 18.226 | 0.737 | 3.546 |
| A1325 | 14.673 | 42.991 | 58.334 | 79.611 | 91.637 | 96.903 | 182.979 | 182.855 | 14.215 | 14.502 | 0.841 | 5.814 | 1.869 | 15.454 | 0.749 | 3.125 |
| A1326 | 13.651 | 41.669 | 57.376 | 78.737 | 89.534 | 94.261 | 178.257 | 177.867 | 18.603 | 18.950 | 0.840 | 6.642 | 1.863 | 17.783 | 0.743 | 3.585 |
| A1327 | 12.940 | 39.012 | 53.704 | 68.459 | 81.236 | 87.003 | 173.758 | 173.645 | 21.673 | 22.008 | 0.868 | 7.473 | 2.024 | 19.364 | 0.727 | 3.701 |
| A1328 | 13.708 | 41.517 | 58.197 | 79.606 | 92.001 | 97.352 | 180.108 | 179.723 | 16.135 | 16.386 | 0.821 | 6.030 | 1.772 | 16.161 | 0.776 | 3.416 |
| A1329 | 14.399 | 46.160 | 62.679 | 81.659 | 94.958 | 100.818 | 180.905 | 180.251 | 20.420 | 20.698 | 0.818 | 6.737 | 1.746 | 18.145 | 0.780 | 3.865 |
| A1330 | 13.409 | 40.985 | 57.252 | 77.706 | 89.444 | 94.549 | 178.356 | 178.293 | 19.202 | 19.479 | 0.838 | 6.710 | 1.847 | 17.905 | 0.756 | 3.646 |
| A1332 | 13.774 | 40.229 | 56.832 | 73.980 | 88.619 | 95.080 | 175.018 | 174.582 | 17.419 | 17.715 | 0.884 | 6.900 | 2.148 | 17.745 | 0.699 | 3.221 |
| A1333 | 14.392 | 42.555 | 58.109 | 78.411 | 90.097 | 95.142 | 178.579 | 178.151 | 15.997 | 16.256 | 0.851 | 6.255 | 1.920 | 16.351 | 0.751 | 3.268 |
| A1334 | 13.413 | 38.132 | 53.502 | 70.982 | 83.891 | 89.552 | 176.196 | 176.236 | 17.947 | 18.225 | 0.840 | 6.512 | 1.857 | 17.195 | 0.762 | 3.517 |
| A1335 | 15.406 | 47.655 | 62.533 | 77.928 | 88.308 | 92.920 | 177.632 | 178.281 | 11.917 | 12.526 | 0.917 | 6.350 | 2.641 | 16.304 | 0.576 | 2.433 |
| A1336 | 13.170 | 43.388 | 62.112 | 81.680 | 92.882 | 97.470 | 175.616 | 175.060 | 15.838 | 16.151 | 0.869 | 6.429 | 2.054 | 16.710 | 0.713 | 3.152 |
| A1337 | 14.714 | 46.219 | 63.188 | 83.353 | 96.553 | 102.253 | 182.656 | 182.308 | 17.294 | 17.673 | 0.874 | 6.821 | 2.116 | 17.733 | 0.702 | 3.260 |
| A1338 | 15.707 | 48.772 | 64.485 | 81.755 | 94.091 | 99.555 | 179.812 | 179.537 | 18.042 | 18.363 | 0.865 | 6.808 | 2.027 | 17.810 | 0.720 | 3.385 |
| A1339 | 13.074 | 40.336 | 54.537 | 70.464 | 80.671 | 85.081 | 171.214 | 171.481 | 20.292 | 20.652 | 0.863 | 7.174 | 1.993 | 18.854 | 0.724 | 3.610 |
| A1340 | 13.960 | 42.460 | 57.555 | 74.594 | 84.793 | 89.117 | 172.895 | 173.129 | 18.042 | 18.360 | 0.854 | 6.678 | 1.940 | 17.453 | 0.744 | 3.451 |
| A1342 | 13.751 | 41.748 | 58.132 | 78.619 | 91.594 | 97.189 | 180.720 | 180.544 | 17.275 | 17.560 | 0.830 | 6.311 | 1.812 | 16.877 | 0.762 | 3.497 |
| A1343 | 13.527 | 42.214 | 58.171 | 74.858 | 87.435 | 92.892 | 177.453 | 177.694 | 18.102 | 18.487 | 0.880 | 7.013 | 2.131 | 18.234 | 0.691 | 3.305 |
| A1345 | 14.219 | 47.305 | 68.016 | 87.796 | 99.644 | 104.603 | 178.967 | 178.754 | 16.019 | 16.311 | 0.855 | 6.294 | 1.947 | 16.447 | 0.743 | 3.250 |
| A1346 | 14.225 | 43.576 | 60.452 | 80.220 | 92.381 | 97.574 | 178.314 | 178.207 | 18.007 | 18.280 | 0.850 | 6.621 | 1.912 | 17.353 | 0.751 | 3.474 |
| A1348 | 14.368 | 44.768 | 61.329 | 78.877 | 90.923 | 96.085 | 177.297 | 177.340 | 14.922 | 15.165 | 0.839 | 5.937 | 1.860 | 15.697 | 0.760 | 3.208 |
| A1349 | 14.519 | 43.808 | 60.727 | 80.949 | 92.650 | 97.613 | 178.247 | 178.035 | 16.928 | 17.172 | 0.827 | 6.203 | 1.785 | 16.522 | 0.779 | 3.480 |
| A1353 | 13.750 | 43.412 | 59.532 | 79.079 | 90.084 | 94.720 | 177.472 | 177.707 | 15.364 | 15.684 | 0.832 | 5.961 | 1.816 | 15.964 | 0.758 | 3.292 |
| A1356 | 14.071 | 42.279 | 57.310 | 75.292 | 85.463 | 89.721 | 174.078 | 174.523 | 17.614 | 18.038 | 0.863 | 6.715 | 2.008 | 17.462 | 0.726 | 3.367 |
| A1358 | 14.454 | 45.265 | 65.303 | 87.991 | 99.957 | 105.142 | 180.741 | 179.906 | 18.863 | 19.162 | 0.822 | 6.516 | 1.767 | 17.566 | 0.768 | 3.697 |
| A1362 | 13.456 | 43.824 | 63.241 | 84.345 | 94.789 | 99.098 | 178.309 | 178.269 | 18.212 | 18.521 | 0.879 | 7.011 | 2.130 | 17.952 | 0.709 | 3.317 |
| A1363 | 14.579 | 44.429 | 61.305 | 82.408 | 94.331 | 99.349 | 179.773 | 179.586 | 20.565 | 20.859 | 0.848 | 7.059 | 1.902 | 18.507 | 0.754 | 3.721 |
| A1364 | 14.123 | 41.812 | 57.704 | 77.181 | 87.599 | 92.075 | 175.543 | 175.690 | 17.993 | 18.345 | 0.836 | 6.501 | 1.845 | 17.367 | 0.750 | 3.542 |
| A1365 | 13.790 | 40.670 | 56.442 | 72.390 | 87.235 | 93.857 | 177.998 | 177.749 | 16.637 | 16.877 | 0.834 | 6.222 | 1.830 | 16.456 | 0.771 | 3.410 |
| A1366 | 14.453 | 43.339 | 61.402 | 84.907 | 96.222 | 100.868 | 181.025 | 180.795 | 18.870 | 19.128 | 0.847 | 6.740 | 1.890 | 17.639 | 0.762 | 3.572 |
| A1367 | 14.235 | 43.835 | 61.014 | 82.640 | 94.119 | 99.092 | 180.273 | 179.786 | 16.549 | 16.822 | 0.838 | 6.244 | 1.857 | 16.546 | 0.758 | 3.382 |
| A1368 | 14.393 | 42.711 | 58.270 | 76.772 | 88.893 | 94.161 | 177.105 | 177.002 | 15.407 | 15.653 | 0.840 | 6.048 | 1.866 | 15.963 | 0.760 | 3.256 |
| A1369 | 14.492 | 44.042 | 60.607 | 81.107 | 92.073 | 96.856 | 175.073 | 174.573 | 17.217 | 17.526 | 0.827 | 6.285 | 1.799 | 16.805 | 0.767 | 3.504 |
| A1370 | 14.613 | 43.653 | 60.133 | 78.155 | 90.515 | 95.806 | 175.986 | 176.033 | 14.559 | 14.809 | 0.815 | 5.706 | 1.767 | 15.478 | 0.768 | 3.263 |
| A1371 | 13.888 | 43.657 | 59.351 | 76.925 | 86.618 | 90.748 | 173.898 | 174.055 | 19.360 | 19.720 | 0.826 | 6.661 | 1.802 | 17.921 | 0.757 | 3.718 |
| A1372 | 13.979 | 43.870 | 59.936 | 79.237 | 90.472 | 95.384 | 177.933 | 177.537 | 16.337 | 16.604 | 0.816 | 6.022 | 1.748 | 16.220 | 0.779 | 3.460 |
| A1374 | 13.851 | 40.290 | 55.720 | 72.986 | 86.106 | 91.953 | 174.431 | 174.448 | 17.200 | 17.459 | 0.823 | 6.224 | 1.770 | 16.641 | 0.780 | 3.525 |
| A1375 | 14.273 | 44.695 | 61.198 | 79.396 | 92.705 | 98.494 | 179.108 | 178.797 | 15.324 | 15.592 | 0.842 | 6.035 | 1.866 | 15.976 | 0.754 | 3.241 |
| A1376 | 14.803 | 43.744 | 58.655 | 78.211 | 89.155 | 93.952 | 176.192 | 175.939 | 18.993 | 19.315 | 0.848 | 6.795 | 1.909 | 18.198 | 0.731 | 3.572 |
| A1377 | 13.848 | 42.578 | 58.136 | 75.378 | 87.358 | 92.560 | 176.669 | 176.808 | 17.580 | 18.054 | 0.876 | 6.856 | 2.092 | 17.872 | 0.695 | 3.285 |
| A1378 | 15.012 | 45.096 | 60.831 | 76.440 | 88.177 | 93.221 | 175.154 | 175.058 | 15.709 | 15.968 | 0.842 | 6.129 | 1.882 | 16.154 | 0.757 | 3.276 |
| A1379 | 14.081 | 42.209 | 58.252 | 77.787 | 88.483 | 93.065 | 176.678 | 176.608 | 16.148 | 16.397 | 0.812 | 5.963 | 1.734 | 16.074 | 0.784 | 3.457 |
| A1380 | 13.001 | 40.115 | 56.810 | 76.980 | 91.049 | 97.106 | 180.040 | 179.747 | 18.822 | 19.084 | 0.819 | 6.499 | 1.766 | 17.398 | 0.781 | 3.698 |
| A1381 | 13.803 | 45.231 | 60.634 | 77.615 | 89.706 | 94.896 | 176.675 | 176.325 | 17.614 | 17.918 | 0.840 | 6.465 | 1.868 | 17.100 | 0.756 | 3.479 |
| A1382 | 14.035 | 43.797 | 62.417 | 81.790 | 92.673 | 97.194 | 173.011 | 173.012 | 15.177 | 15.497 | 0.872 | 6.328 | 2.074 | 16.414 | 0.710 | 3.069 |
| A1383 | 13.430 | 40.771 | 56.899 | 75.991 | 88.055 | 93.241 | 176.693 | 176.625 | 18.162 | 18.437 | 0.833 | 6.482 | 1.819 | 17.167 | 0.774 | 3.572 |
| A1384 | 14.986 | 46.763 | 63.302 | 84.740 | 96.173 | 101.098 | 180.534 | 180.085 | 16.032 | 16.329 | 0.837 | 6.143 | 1.859 | 16.446 | 0.748 | 3.335 |
| A1385 | 14.143 | 43.643 | 60.434 | 80.671 | 92.114 | 96.989 | 178.335 | 178.184 | 17.718 | 18.002 | 0.828 | 6.361 | 1.791 | 16.951 | 0.775 | 3.558 |
| A1386 | 15.383 | 46.249 | 62.534 | 80.088 | 91.916 | 97.089 | 176.107 | 175.612 | 17.457 | 17.832 | 0.884 | 6.924 | 2.162 | 17.795 | 0.695 | 3.220 |
| A1387 | 14.668 | 43.193 | 58.417 | 73.951 | 85.699 | 90.797 | 173.465 | 173.600 | 18.352 | 18.676 | 0.842 | 6.611 | 1.870 | 17.695 | 0.744 | 3.547 |
| A1388 | 14.413 | 44.306 | 60.012 | 79.313 | 89.305 | 93.677 | 173.852 | 173.672 | 17.085 | 17.437 | 0.869 | 6.658 | 2.039 | 17.204 | 0.725 | 3.278 |
| A1389 | 14.196 | 41.791 | 57.111 | 76.078 | 87.598 | 92.698 | 174.773 | 174.360 | 16.956 | 17.258 | 0.857 | 6.507 | 1.954 | 17.003 | 0.737 | 3.337 |
| A1390 | 14.515 | 42.740 | 57.346 | 74.592 | 85.635 | 90.639 | 172.084 | 171.499 | 18.253 | 18.555 | 0.845 | 6.622 | 1.887 | 17.453 | 0.753 | 3.522 |
| A1391 | 13.992 | 41.644 | 57.049 | 75.316 | 85.691 | 90.132 | 175.398 | 175.648 | 15.637 | 15.933 | 0.838 | 6.067 | 1.850 | 16.144 | 0.753 | 3.294 |
| A1393 | 13.029 | 36.323 | 49.685 | 68.354 | 78.682 | 83.211 | 171.811 | 171.712 | 16.336 | 16.636 | 0.803 | 5.940 | 1.696 | 16.215 | 0.780 | 3.517 |
| A1394 | 12.855 | 37.956 | 52.811 | 70.287 | 82.070 | 87.137 | 174.873 | 175.039 | 16.436 | 16.718 | 0.825 | 6.126 | 1.791 | 16.426 | 0.765 | 3.432 |
| A1395 | 13.948 | 40.852 | 57.022 | 78.289 | 90.377 | 95.676 | 176.311 | 175.797 | 16.561 | 16.816 | 0.836 | 6.220 | 1.834 | 16.454 | 0.768 | 3.398 |
| A1396 | 13.121 | 39.756 | 54.301 | 70.240 | 81.527 | 86.494 | 172.517 | 172.575 | 13.779 | 14.064 | 0.825 | 5.609 | 1.794 | 15.097 | 0.759 | 3.143 |
| A1397 | 14.576 | 44.885 | 60.310 | 78.094 | 88.223 | 92.694 | 173.915 | 173.487 | 21.046 | 21.400 | 0.837 | 7.029 | 1.844 | 18.665 | 0.759 | 3.825 |
| A1398 | 13.879 | 40.496 | 55.323 | 74.060 | 85.255 | 90.222 | 172.912 | 172.531 | 16.424 | 16.741 | 0.854 | 6.361 | 1.938 | 16.677 | 0.741 | 3.297 |
| A1399 | 14.901 | 44.965 | 61.174 | 80.869 | 92.649 | 97.747 | 177.890 | 177.556 | 19.320 | 19.651 | 0.855 | 6.914 | 1.944 | 18.088 | 0.741 | 3.568 |
| A1400 | 14.718 | 46.903 | 62.515 | 81.546 | 92.565 | 97.469 | 178.139 | 177.621 | 14.343 | 14.630 | 0.839 | 5.809 | 1.848 | 15.448 | 0.754 | 3.151 |
| A1401 | 14.746 | 44.007 | 59.985 | 79.909 | 91.076 | 95.840 | 176.193 | 175.732 | 18.853 | 19.145 | 0.823 | 6.535 | 1.782 | 17.503 | 0.773 | 3.685 |
| A1403 | 13.768 | 40.926 | 57.836 | 81.018 | 92.712 | 97.685 | 180.766 | 180.314 | 16.490 | 16.774 | 0.850 | 6.336 | 1.907 | 16.805 | 0.739 | 3.330 |
| A1405 | 13.921 | 43.740 | 60.536 | 77.112 | 90.403 | 96.178 | 178.836 | 178.881 | 15.902 | 16.216 | 0.855 | 6.298 | 1.964 | 16.482 | 0.736 | 3.231 |
| A1406 | 13.020 | 37.817 | 53.018 | 71.397 | 83.249 | 88.428 | 175.090 | 175.212 | 18.085 | 18.367 | 0.838 | 6.510 | 1.840 | 17.239 | 0.764 | 3.544 |
| A1407 | 13.828 | 44.320 | 60.697 | 77.306 | 88.014 | 92.520 | 174.638 | 174.728 | 17.461 | 17.809 | 0.858 | 6.642 | 1.987 | 17.526 | 0.721 | 3.366 |
| A1408 | 13.243 | 37.642 | 52.289 | 70.497 | 81.944 | 86.892 | 172.570 | 172.838 | 17.429 | 17.757 | 0.853 | 6.567 | 1.947 | 17.184 | 0.740 | 3.393 |
| A1410 | 13.424 | 41.939 | 57.176 | 74.924 | 85.712 | 90.402 | 175.226 | 175.133 | 14.287 | 14.590 | 0.853 | 5.957 | 1.969 | 15.742 | 0.725 | 3.061 |
| A1411 | 14.261 | 44.326 | 59.669 | 77.166 | 88.826 | 93.888 | 178.871 | 179.061 | 14.139 | 14.432 | 0.840 | 5.786 | 1.873 | 15.394 | 0.747 | 3.118 |
| A1413 | 14.473 | 42.918 | 59.826 | 81.769 | 92.656 | 97.228 | 179.028 | 179.098 | 17.409 | 17.690 | 0.821 | 6.241 | 1.760 | 16.758 | 0.777 | 3.557 |
| A1415 | 14.683 | 43.652 | 58.944 | 78.514 | 90.639 | 96.067 | 179.447 | 179.028 | 16.935 | 17.250 | 0.812 | 6.104 | 1.725 | 16.617 | 0.772 | 3.546 |
| A1416 | 14.892 | 45.448 | 61.102 | 79.277 | 90.637 | 95.631 | 177.560 | 177.457 | 18.052 | 18.376 | 0.816 | 6.358 | 1.762 | 17.200 | 0.767 | 3.633 |
| A1417 | 14.300 | 45.859 | 61.277 | 79.525 | 90.640 | 95.464 | 178.271 | 178.092 | 17.118 | 17.465 | 0.849 | 6.465 | 1.922 | 17.107 | 0.735 | 3.383 |
| A1418 | 13.298 | 39.554 | 54.584 | 71.308 | 83.235 | 88.384 | 173.646 | 173.713 | 17.308 | 17.605 | 0.815 | 6.220 | 1.753 | 16.796 | 0.771 | 3.563 |
| A1419 | 13.255 | 39.612 | 55.328 | 72.766 | 85.390 | 90.897 | 176.069 | 176.094 | 18.117 | 18.388 | 0.854 | 6.684 | 1.939 | 17.421 | 0.749 | 3.459 |
| A1420 | 13.542 | 39.419 | 55.701 | 77.488 | 90.690 | 96.421 | 178.165 | 177.473 | 18.653 | 18.931 | 0.843 | 6.675 | 1.878 | 17.561 | 0.760 | 3.568 |
| A1421 | 14.517 | 44.238 | 60.111 | 80.830 | 92.920 | 98.106 | 180.742 | 180.326 | 14.129 | 14.378 | 0.858 | 5.934 | 1.957 | 15.485 | 0.740 | 3.039 |
| A1424 | 14.312 | 41.396 | 57.325 | 78.512 | 90.061 | 95.034 | 176.801 | 176.325 | 16.459 | 16.761 | 0.847 | 6.312 | 1.902 | 16.715 | 0.743 | 3.337 |
| A1426 | 13.920 | 42.598 | 58.558 | 74.947 | 87.277 | 92.681 | 174.405 | 174.232 | 16.999 | 17.312 | 0.884 | 6.834 | 2.151 | 17.491 | 0.702 | 3.185 |
| A1427 | 15.453 | 42.462 | 56.739 | 75.064 | 86.088 | 90.899 | 175.363 | 175.402 | 15.121 | 15.378 | 0.852 | 6.104 | 1.936 | 15.956 | 0.746 | 3.169 |
| A1428 | 13.780 | 42.266 | 59.454 | 80.092 | 92.505 | 97.788 | 178.529 | 178.501 | 15.957 | 16.218 | 0.849 | 6.220 | 1.906 | 16.316 | 0.753 | 3.273 |
| A1429 | 13.752 | 44.456 | 64.275 | 84.022 | 95.634 | 100.612 | 178.174 | 177.783 | 16.781 | 17.035 | 0.847 | 6.361 | 1.894 | 16.791 | 0.752 | 3.369 |
| A1430 | 14.635 | 46.633 | 64.141 | 84.278 | 97.305 | 102.936 | 181.033 | 180.403 | 16.795 | 17.070 | 0.846 | 6.362 | 1.902 | 16.718 | 0.753 | 3.367 |
| A1431 | 13.779 | 39.828 | 57.270 | 79.348 | 91.305 | 96.428 | 178.402 | 178.301 | 15.875 | 16.143 | 0.835 | 6.084 | 1.830 | 16.157 | 0.764 | 3.333 |
| A1432 | 14.231 | 42.453 | 58.760 | 76.979 | 89.890 | 95.526 | 179.703 | 179.905 | 15.254 | 15.516 | 0.830 | 5.928 | 1.812 | 15.938 | 0.759 | 3.285 |
| A1433 | 12.712 | 38.184 | 54.626 | 72.360 | 85.017 | 90.465 | 176.179 | 175.910 | 14.578 | 14.825 | 0.830 | 5.799 | 1.817 | 15.452 | 0.766 | 3.211 |
| A1434 | 13.051 | 40.854 | 56.732 | 74.535 | 85.195 | 89.796 | 174.547 | 174.992 | 20.172 | 20.507 | 0.860 | 7.123 | 1.973 | 18.560 | 0.735 | 3.619 |
| A1435 | 12.553 | 38.879 | 54.613 | 71.924 | 84.096 | 89.445 | 174.044 | 174.090 | 20.302 | 20.664 | 0.846 | 6.991 | 1.894 | 18.700 | 0.734 | 3.708 |
| A1440 | 13.926 | 48.149 | 70.326 | 91.753 | 104.685 | 110.136 | 182.532 | 181.788 | 19.502 | 19.807 | 0.836 | 6.757 | 1.837 | 17.930 | 0.762 | 3.688 |
| A1441 | 13.548 | 38.320 | 51.892 | 65.430 | 78.010 | 83.624 | 170.680 | 170.779 | 16.917 | 17.216 | 0.851 | 6.432 | 1.921 | 16.827 | 0.750 | 3.362 |
| A1443 | 14.189 | 45.434 | 61.611 | 80.899 | 91.825 | 96.404 | 177.807 | 178.100 | 15.534 | 15.859 | 0.848 | 6.144 | 1.906 | 16.234 | 0.741 | 3.234 |
| A1444 | 14.320 | 39.579 | 54.936 | 73.045 | 84.890 | 89.956 | 173.004 | 172.933 | 17.055 | 17.388 | 0.855 | 6.509 | 1.954 | 17.281 | 0.726 | 3.347 |
| A1445 | 14.761 | 45.819 | 62.233 | 80.763 | 91.460 | 96.019 | 177.549 | 177.538 | 18.916 | 19.229 | 0.848 | 6.764 | 1.900 | 17.775 | 0.752 | 3.571 |
| A1447 | 15.493 | 46.956 | 63.598 | 84.417 | 96.933 | 102.385 | 181.931 | 181.383 | 18.413 | 18.729 | 0.854 | 6.755 | 1.944 | 17.618 | 0.746 | 3.486 |
| A1448 | 14.165 | 46.361 | 65.791 | 86.618 | 96.188 | 100.148 | 175.706 | 175.334 | 17.337 | 17.652 | 0.834 | 6.353 | 1.827 | 16.958 | 0.757 | 3.486 |
| A1450 | 15.976 | 47.888 | 64.399 | 84.325 | 95.923 | 100.937 | 179.298 | 178.697 | 18.003 | 18.310 | 0.859 | 6.716 | 1.965 | 17.464 | 0.741 | 3.425 |
| A1453 | 13.507 | 39.831 | 55.550 | 74.145 | 84.518 | 89.020 | 173.158 | 173.334 | 16.846 | 17.157 | 0.873 | 6.654 | 2.064 | 17.165 | 0.718 | 3.237 |
| A1454 | 14.308 | 44.402 | 61.110 | 82.066 | 93.655 | 98.681 | 180.156 | 179.789 | 16.129 | 16.460 | 0.866 | 6.439 | 2.022 | 16.674 | 0.728 | 3.200 |
| A1455 | 13.882 | 40.069 | 54.473 | 73.054 | 84.696 | 89.757 | 177.449 | 177.460 | 14.895 | 15.174 | 0.849 | 6.009 | 1.908 | 15.806 | 0.747 | 3.163 |
| A1456 | 14.269 | 40.271 | 56.231 | 76.970 | 88.743 | 93.825 | 177.274 | 177.441 | 17.859 | 18.142 | 0.829 | 6.403 | 1.805 | 17.103 | 0.767 | 3.562 |
| A1457 | 14.671 | 43.066 | 59.094 | 79.052 | 91.064 | 96.221 | 180.927 | 180.978 | 17.110 | 17.417 | 0.855 | 6.513 | 1.951 | 17.020 | 0.742 | 3.355 |
| A1458 | 14.353 | 41.808 | 56.415 | 75.022 | 85.921 | 90.726 | 174.196 | 174.235 | 14.399 | 14.676 | 0.851 | 5.934 | 1.924 | 15.538 | 0.748 | 3.099 |
| A1460 | 13.769 | 39.548 | 54.067 | 68.814 | 80.759 | 85.944 | 175.764 | 176.873 | 14.873 | 15.288 | 0.910 | 6.831 | 2.467 | 16.926 | 0.652 | 2.797 |
| A1462 | 13.660 | 39.912 | 54.578 | 75.156 | 85.945 | 90.661 | 175.471 | 175.102 | 16.517 | 16.794 | 0.847 | 6.314 | 1.899 | 16.588 | 0.754 | 3.339 |
| A1463 | 13.623 | 41.999 | 58.094 | 78.570 | 90.344 | 95.457 | 180.348 | 179.960 | 16.237 | 16.547 | 0.874 | 6.557 | 2.082 | 16.870 | 0.716 | 3.165 |
| A1464 | 14.560 | 43.136 | 59.747 | 78.970 | 91.822 | 97.343 | 179.369 | 179.256 | 15.523 | 15.793 | 0.875 | 6.424 | 2.093 | 16.482 | 0.717 | 3.088 |
| A1466 | 13.268 | 38.672 | 52.873 | 67.441 | 77.938 | 82.554 | 172.429 | 172.999 | 14.706 | 15.059 | 0.856 | 6.065 | 1.973 | 16.113 | 0.715 | 3.101 |
| A1467 | 13.588 | 41.509 | 57.372 | 73.994 | 87.603 | 93.663 | 178.214 | 178.363 | 17.969 | 18.273 | 0.814 | 6.308 | 1.740 | 17.290 | 0.765 | 3.640 |
| A1468 | 14.022 | 42.437 | 57.537 | 76.085 | 86.804 | 91.621 | 174.082 | 173.876 | 16.444 | 16.735 | 0.820 | 6.079 | 1.766 | 16.363 | 0.771 | 3.456 |
| A1469 | 13.032 | 40.155 | 56.566 | 75.451 | 88.544 | 94.246 | 176.521 | 176.335 | 17.610 | 17.978 | 0.876 | 6.870 | 2.110 | 17.993 | 0.690 | 3.280 |
| A1471 | 13.406 | 38.801 | 53.785 | 73.022 | 84.297 | 89.229 | 173.769 | 173.495 | 14.014 | 14.278 | 0.830 | 5.702 | 1.827 | 15.202 | 0.762 | 3.144 |
| A1472 | 13.735 | 44.785 | 61.120 | 81.644 | 93.233 | 98.272 | 180.653 | 180.472 | 16.930 | 17.413 | 0.815 | 6.191 | 1.775 | 16.887 | 0.754 | 3.526 |
| A1473 | 13.472 | 41.332 | 56.212 | 74.840 | 86.779 | 92.039 | 177.249 | 176.850 | 14.592 | 14.868 | 0.832 | 5.808 | 1.819 | 15.530 | 0.759 | 3.206 |
| A1476 | 14.031 | 45.553 | 66.219 | 89.443 | 101.735 | 107.099 | 180.471 | 179.515 | 19.304 | 19.601 | 0.846 | 6.806 | 1.889 | 17.880 | 0.757 | 3.614 |
| A1477 | 14.229 | 46.628 | 65.579 | 83.695 | 95.774 | 100.895 | 176.778 | 176.334 | 18.378 | 18.651 | 0.840 | 6.583 | 1.851 | 17.362 | 0.766 | 3.559 |
| A1478 | 13.510 | 43.225 | 62.940 | 82.660 | 95.631 | 101.317 | 177.533 | 176.920 | 15.843 | 16.091 | 0.816 | 5.937 | 1.748 | 15.948 | 0.782 | 3.408 |
| A1479 | 13.421 | 43.403 | 64.907 | 88.513 | 99.382 | 103.956 | 180.910 | 180.763 | 15.105 | 15.428 | 0.855 | 6.144 | 1.974 | 16.151 | 0.727 | 3.147 |
| A1480 | 13.402 | 39.183 | 54.092 | 73.523 | 85.380 | 90.595 | 173.790 | 173.571 | 18.704 | 19.003 | 0.829 | 6.570 | 1.816 | 17.481 | 0.768 | 3.635 |
| A1481 | 14.956 | 50.675 | 75.183 | 97.556 | 110.450 | 115.763 | 185.438 | 184.924 | 17.979 | 18.244 | 0.856 | 6.674 | 1.950 | 17.370 | 0.748 | 3.437 |
| A1482 | 13.451 | 40.086 | 55.503 | 76.631 | 89.615 | 95.354 | 179.161 | 178.417 | 16.465 | 16.708 | 0.812 | 6.011 | 1.724 | 16.180 | 0.790 | 3.494 |
| A1484 | 14.292 | 41.578 | 56.827 | 77.898 | 88.082 | 92.539 | 175.012 | 174.493 | 16.370 | 16.645 | 0.861 | 6.427 | 1.986 | 16.795 | 0.733 | 3.252 |
| A1485 | 14.374 | 40.953 | 55.266 | 73.650 | 85.156 | 90.291 | 176.213 | 176.094 | 17.039 | 17.313 | 0.829 | 6.259 | 1.806 | 16.669 | 0.771 | 3.478 |
| A1486 | 14.693 | 48.143 | 65.759 | 87.225 | 98.070 | 102.660 | 178.250 | 177.238 | 17.675 | 17.952 | 0.824 | 6.339 | 1.787 | 16.935 | 0.774 | 3.564 |
| A1487 | 14.904 | 43.471 | 58.208 | 76.421 | 87.521 | 92.337 | 176.257 | 176.037 | 14.956 | 15.255 | 0.837 | 5.963 | 1.866 | 15.825 | 0.750 | 3.216 |
| A1488 | 14.875 | 43.700 | 59.005 | 78.506 | 90.038 | 95.022 | 178.583 | 178.331 | 14.801 | 15.118 | 0.868 | 6.209 | 2.048 | 16.113 | 0.716 | 3.054 |
| A1490 | 13.104 | 39.961 | 55.635 | 76.319 | 86.858 | 91.271 | 174.822 | 174.741 | 17.365 | 17.666 | 0.811 | 6.192 | 1.730 | 16.806 | 0.772 | 3.596 |
| A1491 | 13.763 | 40.428 | 55.047 | 72.956 | 83.216 | 87.550 | 172.906 | 173.121 | 18.079 | 18.386 | 0.854 | 6.675 | 1.940 | 17.840 | 0.727 | 3.459 |
| A1492 | 15.248 | 45.693 | 60.880 | 80.823 | 91.471 | 96.083 | 178.286 | 177.949 | 15.310 | 15.566 | 0.806 | 5.780 | 1.715 | 15.694 | 0.781 | 3.388 |
| A1493 | 14.298 | 41.566 | 57.366 | 78.157 | 89.324 | 93.987 | 177.773 | 177.691 | 18.652 | 18.927 | 0.844 | 6.681 | 1.882 | 17.575 | 0.758 | 3.561 |
| A1494 | 14.044 | 41.630 | 56.698 | 75.211 | 86.556 | 91.462 | 176.702 | 176.566 | 16.061 | 16.366 | 0.855 | 6.324 | 1.957 | 16.515 | 0.740 | 3.249 |
| A1495 | 13.079 | 37.342 | 51.579 | 70.042 | 82.492 | 87.961 | 176.454 | 176.320 | 11.471 | 11.695 | 0.839 | 5.209 | 1.865 | 13.841 | 0.754 | 2.812 |
| A1496 | 15.290 | 45.224 | 60.411 | 79.483 | 90.780 | 95.659 | 178.928 | 178.611 | 16.875 | 17.138 | 0.813 | 6.114 | 1.745 | 16.476 | 0.781 | 3.526 |
| A1497 | 14.895 | 45.462 | 60.281 | 75.084 | 86.093 | 90.918 | 173.639 | 173.992 | 13.661 | 13.923 | 0.846 | 5.747 | 1.898 | 15.312 | 0.739 | 3.041 |
| A1498 | 13.056 | 40.896 | 55.917 | 71.276 | 84.847 | 90.740 | 176.285 | 176.340 | 15.398 | 15.671 | 0.849 | 6.132 | 1.917 | 16.263 | 0.738 | 3.213 |
| A1499 | 13.971 | 44.851 | 60.417 | 79.025 | 90.799 | 95.944 | 176.704 | 176.159 | 16.534 | 16.819 | 0.838 | 6.239 | 1.852 | 16.550 | 0.757 | 3.380 |
| A1500 | 14.328 | 45.710 | 61.655 | 81.622 | 93.291 | 98.399 | 178.389 | 177.845 | 16.758 | 17.022 | 0.815 | 6.096 | 1.742 | 16.409 | 0.782 | 3.511 |
| A1501 | 13.005 | 39.894 | 55.146 | 72.468 | 82.604 | 86.952 | 172.618 | 172.950 | 17.621 | 17.940 | 0.819 | 6.282 | 1.763 | 16.939 | 0.770 | 3.580 |
| A1502 | 14.553 | 42.392 | 58.840 | 78.908 | 90.281 | 95.182 | 180.823 | 181.230 | 16.663 | 16.929 | 0.845 | 6.323 | 1.883 | 16.602 | 0.759 | 3.366 |
| A1503 | 12.891 | 36.101 | 51.221 | 71.448 | 82.069 | 86.523 | 174.289 | 174.621 | 18.032 | 18.349 | 0.859 | 6.724 | 1.971 | 17.474 | 0.741 | 3.425 |
| A1504 | 14.238 | 41.556 | 57.066 | 78.396 | 89.987 | 95.062 | 176.904 | 176.280 | 17.568 | 17.877 | 0.826 | 6.320 | 1.783 | 16.985 | 0.765 | 3.553 |
| A1505 | 13.529 | 40.106 | 56.525 | 78.970 | 90.766 | 95.712 | 178.077 | 177.842 | 17.454 | 17.746 | 0.833 | 6.369 | 1.829 | 16.961 | 0.762 | 3.501 |
| A1506 | 13.263 | 37.582 | 52.057 | 68.242 | 79.495 | 84.385 | 171.537 | 171.626 | 15.121 | 15.385 | 0.841 | 5.994 | 1.869 | 15.813 | 0.758 | 3.221 |
| A1507 | 14.235 | 44.253 | 59.127 | 76.032 | 86.540 | 91.170 | 173.650 | 173.221 | 18.529 | 18.902 | 0.876 | 7.020 | 2.094 | 18.138 | 0.706 | 3.366 |
| A1508 | 15.068 | 45.348 | 61.002 | 81.919 | 93.391 | 98.358 | 180.156 | 179.721 | 17.002 | 17.260 | 0.815 | 6.149 | 1.752 | 16.525 | 0.781 | 3.532 |
| A1509 | 15.387 | 47.346 | 66.024 | 88.987 | 102.913 | 108.663 | 183.006 | 182.602 | 18.241 | 18.509 | 0.845 | 6.614 | 1.889 | 17.407 | 0.755 | 3.518 |
| A1513 | 13.126 | 40.738 | 55.106 | 71.569 | 83.454 | 88.724 | 174.628 | 174.552 | 15.575 | 15.869 | 0.817 | 5.899 | 1.757 | 16.110 | 0.759 | 3.375 |
| A1514 | 13.889 | 42.256 | 57.357 | 75.705 | 86.287 | 90.775 | 174.171 | 174.110 | 16.789 | 17.093 | 0.857 | 6.478 | 1.963 | 16.914 | 0.737 | 3.315 |
| A1515 | 13.681 | 44.209 | 59.483 | 76.224 | 87.815 | 92.842 | 176.667 | 176.715 | 17.855 | 18.184 | 0.836 | 6.456 | 1.832 | 17.245 | 0.754 | 3.533 |
| A1516 | 13.465 | 39.304 | 53.707 | 69.528 | 82.264 | 87.885 | 173.494 | 173.447 | 18.415 | 18.705 | 0.840 | 6.601 | 1.864 | 17.454 | 0.757 | 3.555 |
| A1517 | 14.085 | 39.619 | 53.301 | 71.475 | 83.432 | 88.689 | 175.731 | 175.390 | 15.659 | 15.910 | 0.839 | 6.086 | 1.858 | 16.066 | 0.762 | 3.286 |
| A1518 | 14.191 | 40.924 | 54.901 | 71.936 | 82.337 | 86.936 | 171.405 | 171.405 | 15.909 | 16.248 | 0.845 | 6.210 | 1.907 | 16.415 | 0.741 | 3.275 |
| A1521 | 13.604 | 41.494 | 56.532 | 72.067 | 84.936 | 90.655 | 176.686 | 176.740 | 16.037 | 16.349 | 0.843 | 6.190 | 1.879 | 16.434 | 0.745 | 3.310 |
| A1522 | 12.806 | 41.679 | 57.749 | 77.548 | 89.331 | 94.457 | 179.046 | 178.911 | 19.814 | 20.117 | 0.846 | 6.904 | 1.887 | 18.147 | 0.755 | 3.670 |
| A1523 | 13.226 | 40.458 | 56.375 | 73.685 | 88.082 | 94.381 | 179.096 | 179.099 | 16.512 | 16.821 | 0.870 | 6.570 | 2.047 | 16.985 | 0.719 | 3.223 |
| A1524 | 14.200 | 42.739 | 58.044 | 76.313 | 86.659 | 91.067 | 174.891 | 175.199 | 17.793 | 18.135 | 0.856 | 6.661 | 1.955 | 17.699 | 0.724 | 3.422 |
| A1525 | 13.172 | 39.247 | 53.271 | 69.835 | 79.343 | 83.417 | 169.658 | 170.121 | 15.281 | 15.585 | 0.797 | 5.709 | 1.676 | 15.658 | 0.782 | 3.422 |
| A1526 | 14.423 | 44.692 | 60.842 | 79.726 | 91.076 | 96.014 | 176.666 | 176.131 | 19.965 | 20.285 | 0.855 | 7.015 | 1.940 | 18.326 | 0.745 | 3.630 |
| A1527 | 15.030 | 47.363 | 64.277 | 83.626 | 94.260 | 98.636 | 177.499 | 177.457 | 19.805 | 20.092 | 0.812 | 6.617 | 1.738 | 17.824 | 0.783 | 3.823 |
| A1528 | 14.281 | 44.714 | 60.368 | 79.081 | 90.819 | 95.963 | 176.193 | 175.693 | 22.707 | 23.088 | 0.845 | 7.395 | 1.893 | 19.851 | 0.734 | 3.925 |
| A1529 | 14.118 | 40.624 | 55.558 | 71.967 | 84.128 | 89.467 | 174.153 | 174.635 | 17.571 | 17.853 | 0.829 | 6.358 | 1.812 | 16.929 | 0.769 | 3.529 |
| A1530 | 12.954 | 38.925 | 55.337 | 75.144 | 86.916 | 91.963 | 174.977 | 174.840 | 17.459 | 17.756 | 0.839 | 6.413 | 1.848 | 17.042 | 0.756 | 3.478 |
| A1531 | 14.710 | 45.835 | 62.207 | 81.479 | 93.828 | 99.198 | 180.706 | 180.455 | 13.858 | 14.129 | 0.871 | 6.026 | 2.060 | 15.515 | 0.723 | 2.940 |
| A1532 | 13.699 | 41.352 | 57.446 | 75.367 | 88.219 | 93.827 | 175.500 | 175.331 | 17.403 | 17.668 | 0.810 | 6.172 | 1.721 | 16.671 | 0.786 | 3.599 |
| A1533 | 13.849 | 40.917 | 56.662 | 75.395 | 85.235 | 89.499 | 170.333 | 170.290 | 17.426 | 17.735 | 0.825 | 6.285 | 1.784 | 16.858 | 0.769 | 3.538 |
| A1534 | 14.196 | 43.377 | 59.586 | 77.708 | 89.711 | 94.873 | 176.153 | 176.152 | 18.565 | 18.868 | 0.864 | 6.884 | 2.003 | 17.740 | 0.740 | 3.447 |
| A1535 | 14.509 | 45.622 | 62.063 | 81.763 | 94.178 | 99.575 | 180.321 | 179.982 | 14.614 | 14.884 | 0.841 | 5.910 | 1.880 | 15.613 | 0.753 | 3.164 |
| A1536 | 14.225 | 40.115 | 53.445 | 69.572 | 78.893 | 82.966 | 170.615 | 171.359 | 11.865 | 12.214 | 0.836 | 5.298 | 1.861 | 14.293 | 0.731 | 2.873 |
| A1537 | 13.087 | 40.724 | 55.778 | 74.138 | 86.268 | 91.520 | 175.424 | 174.849 | 17.375 | 17.634 | 0.775 | 5.944 | 1.598 | 16.435 | 0.808 | 3.733 |
| A1539 | 13.726 | 41.813 | 57.607 | 75.689 | 88.071 | 93.436 | 176.578 | 176.602 | 15.991 | 16.291 | 0.859 | 6.337 | 1.968 | 16.615 | 0.728 | 3.228 |
| A1540 | 13.979 | 41.599 | 56.621 | 73.818 | 83.557 | 87.690 | 172.220 | 172.301 | 16.776 | 17.075 | 0.849 | 6.383 | 1.908 | 16.828 | 0.744 | 3.355 |
| A1543 | 13.182 | 38.534 | 52.111 | 66.338 | 77.214 | 81.929 | 170.545 | 170.743 | 17.957 | 18.229 | 0.873 | 6.874 | 2.070 | 17.612 | 0.727 | 3.335 |
| A1544 | 15.324 | 45.811 | 61.763 | 81.511 | 92.217 | 96.866 | 176.273 | 175.980 | 15.589 | 15.881 | 0.838 | 6.060 | 1.852 | 16.109 | 0.754 | 3.287 |
| A1546 | 13.357 | 39.027 | 53.872 | 70.407 | 83.750 | 89.723 | 176.809 | 176.996 | 19.752 | 20.054 | 0.847 | 6.910 | 1.896 | 18.131 | 0.756 | 3.656 |
| A1547 | 13.300 | 38.161 | 53.999 | 75.306 | 88.069 | 93.563 | 178.874 | 178.969 | 17.501 | 17.767 | 0.812 | 6.198 | 1.728 | 16.739 | 0.784 | 3.600 |
| A1548 | 13.556 | 42.161 | 58.607 | 77.887 | 88.485 | 93.067 | 177.795 | 178.302 | 15.561 | 15.881 | 0.859 | 6.257 | 1.983 | 16.354 | 0.730 | 3.179 |
| A1550 | 14.553 | 45.223 | 60.889 | 75.393 | 89.364 | 95.567 | 176.596 | 176.331 | 18.250 | 18.546 | 0.865 | 6.827 | 2.009 | 17.658 | 0.734 | 3.411 |
| A1551 | 13.012 | 36.823 | 51.856 | 70.322 | 83.599 | 89.477 | 178.424 | 178.693 | 16.380 | 16.658 | 0.896 | 6.875 | 2.269 | 17.249 | 0.691 | 3.043 |
| A1552 | 14.325 | 44.920 | 62.745 | 82.127 | 92.712 | 97.287 | 171.793 | 171.302 | 18.845 | 19.168 | 0.825 | 6.543 | 1.781 | 17.571 | 0.767 | 3.682 |
| A1553 | 13.774 | 39.802 | 55.432 | 73.251 | 86.596 | 92.454 | 175.329 | 175.079 | 18.143 | 18.440 | 0.867 | 6.822 | 2.017 | 17.618 | 0.733 | 3.392 |
| A1554 | 14.920 | 46.307 | 62.629 | 83.883 | 95.429 | 100.414 | 180.208 | 179.608 | 18.399 | 18.673 | 0.848 | 6.670 | 1.903 | 17.586 | 0.750 | 3.519 |
| A1555 | 14.438 | 40.123 | 54.522 | 73.665 | 85.843 | 91.246 | 176.722 | 176.518 | 16.272 | 16.538 | 0.808 | 5.962 | 1.718 | 16.126 | 0.786 | 3.483 |
| A2087 | 13.628 | 39.605 | 55.708 | 76.789 | 89.560 | 95.108 | 177.512 | 176.969 | 14.718 | 14.991 | 0.854 | 6.051 | 1.954 | 15.810 | 0.740 | 3.112 |
| A2135 | 13.312 | 40.523 | 55.425 | 70.726 | 85.528 | 92.191 | 177.064 | 176.839 | 15.833 | 16.118 | 0.821 | 5.984 | 1.772 | 16.103 | 0.768 | 3.388 |
| A2138 | 14.238 | 43.186 | 59.540 | 79.578 | 90.311 | 94.837 | 176.817 | 176.767 | 18.401 | 18.670 | 0.866 | 6.862 | 2.007 | 17.685 | 0.739 | 3.426 |
| A2166 | 14.282 | 44.905 | 60.617 | 80.348 | 91.679 | 96.663 | 176.458 | 175.611 | 16.963 | 17.249 | 0.822 | 6.198 | 1.782 | 16.699 | 0.768 | 3.497 |
| A2281 | 13.922 | 40.908 | 55.187 | 74.343 | 84.567 | 89.084 | 173.011 | 172.963 | 18.307 | 18.621 | 0.858 | 6.768 | 1.971 | 17.602 | 0.741 | 3.451 |
| A2284 | 13.928 | 41.666 | 57.776 | 78.137 | 89.713 | 94.715 | 177.948 | 178.135 | 15.656 | 15.917 | 0.835 | 6.036 | 1.830 | 16.049 | 0.764 | 3.310 |
| A2344 | 14.596 | 45.772 | 62.735 | 83.054 | 93.385 | 97.888 | 176.229 | 175.910 | 14.644 | 14.943 | 0.868 | 6.156 | 2.026 | 15.975 | 0.721 | 3.045 |
| A2409 | 14.260 | 43.232 | 57.820 | 75.437 | 85.127 | 89.342 | 172.893 | 172.727 | 18.210 | 18.543 | 0.862 | 6.798 | 1.999 | 17.717 | 0.729 | 3.420 |
| A2442 | 13.755 | 40.474 | 55.510 | 74.363 | 85.413 | 90.217 | 174.723 | 174.367 | 15.921 | 16.230 | 0.841 | 6.152 | 1.873 | 16.312 | 0.751 | 3.305 |
| C080 | 13.629 | 38.406 | 54.688 | 77.739 | 91.183 | 96.959 | 180.721 | 180.243 | 16.671 | 16.921 | 0.826 | 6.166 | 1.796 | 16.493 | 0.771 | 3.453 |
| C081 | 15.682 | 45.307 | 61.174 | 82.144 | 94.600 | 100.035 | 180.112 | 179.457 | 17.427 | 17.702 | 0.840 | 6.428 | 1.862 | 16.981 | 0.760 | 3.465 |
| C082 | 14.462 | 41.722 | 57.299 | 76.923 | 88.917 | 94.268 | 177.718 | 177.536 | 18.881 | 19.166 | 0.860 | 6.880 | 1.972 | 17.841 | 0.744 | 3.502 |
| C083 | 15.173 | 45.904 | 61.743 | 80.834 | 92.450 | 97.495 | 178.712 | 178.408 | 16.366 | 16.641 | 0.835 | 6.182 | 1.839 | 16.444 | 0.760 | 3.378 |
| C084 | 14.916 | 45.584 | 60.693 | 77.896 | 88.998 | 93.863 | 174.377 | 174.177 | 17.360 | 17.691 | 0.894 | 7.036 | 2.244 | 17.989 | 0.678 | 3.148 |
| C087 | 13.797 | 41.643 | 56.745 | 73.987 | 84.609 | 89.243 | 173.249 | 173.259 | 16.532 | 16.830 | 0.862 | 6.479 | 1.997 | 16.847 | 0.731 | 3.261 |
| C089 | 13.851 | 40.325 | 56.158 | 75.044 | 88.130 | 93.843 | 176.152 | 175.829 | 20.587 | 20.865 | 0.824 | 6.828 | 1.782 | 18.198 | 0.780 | 3.843 |
| C091 | 13.702 | 39.870 | 54.837 | 74.266 | 84.502 | 88.886 | 172.645 | 172.523 | 19.002 | 19.330 | 0.835 | 6.675 | 1.841 | 17.820 | 0.753 | 3.640 |
| C092 | 14.835 | 44.017 | 59.695 | 78.919 | 89.855 | 94.578 | 175.683 | 175.550 | 17.561 | 17.829 | 0.834 | 6.387 | 1.824 | 16.981 | 0.765 | 3.509 |
| C093 | 14.666 | 42.186 | 58.291 | 79.613 | 91.053 | 95.884 | 176.854 | 176.656 | 17.224 | 17.460 | 0.812 | 6.153 | 1.727 | 16.523 | 0.793 | 3.573 |
| C094 | 14.274 | 39.667 | 54.935 | 71.406 | 81.066 | 85.323 | 171.599 | 173.472 | 15.893 | 16.304 | 0.876 | 6.543 | 2.123 | 17.100 | 0.687 | 3.114 |
| C096 | 13.759 | 40.137 | 55.715 | 74.865 | 85.161 | 89.539 | 174.175 | 174.406 | 18.875 | 19.205 | 0.874 | 7.052 | 2.076 | 18.111 | 0.721 | 3.416 |
| C097 | 14.097 | 40.960 | 55.575 | 72.849 | 82.216 | 86.319 | 169.872 | 169.948 | 16.814 | 17.083 | 0.867 | 6.568 | 2.022 | 16.960 | 0.733 | 3.264 |
| C098 | 14.034 | 42.246 | 57.933 | 77.474 | 89.045 | 94.045 | 178.408 | 178.539 | 17.366 | 17.673 | 0.846 | 6.477 | 1.899 | 17.099 | 0.746 | 3.428 |
| C100 | 14.143 | 42.665 | 57.800 | 76.259 | 87.846 | 92.888 | 177.155 | 177.247 | 16.667 | 16.922 | 0.845 | 6.322 | 1.886 | 16.616 | 0.758 | 3.367 |
| C101 | 13.878 | 43.166 | 59.393 | 78.911 | 91.082 | 96.245 | 178.911 | 178.702 | 19.165 | 19.487 | 0.847 | 6.814 | 1.908 | 17.915 | 0.750 | 3.592 |
| C103 | 13.336 | 39.517 | 54.346 | 72.235 | 84.224 | 89.401 | 175.919 | 176.098 | 17.789 | 18.121 | 0.835 | 6.457 | 1.843 | 17.161 | 0.759 | 3.524 |
| C105 | 12.805 | 37.702 | 54.742 | 74.535 | 87.863 | 93.688 | 177.106 | 176.865 | 15.559 | 15.844 | 0.817 | 5.894 | 1.753 | 15.920 | 0.771 | 3.374 |
| C106 | 13.059 | 36.890 | 52.244 | 69.445 | 82.101 | 87.733 | 174.990 | 175.320 | 17.194 | 17.460 | 0.846 | 6.420 | 1.884 | 16.844 | 0.760 | 3.417 |
| C107 | 14.626 | 44.866 | 61.143 | 78.471 | 90.931 | 96.345 | 175.898 | 175.765 | 19.648 | 19.975 | 0.857 | 6.993 | 1.953 | 18.453 | 0.732 | 3.588 |
| C108 | 13.571 | 39.791 | 56.713 | 77.127 | 90.693 | 96.437 | 179.664 | 179.725 | 16.607 | 16.870 | 0.894 | 6.892 | 2.247 | 17.281 | 0.698 | 3.076 |
| C109 | 13.452 | 41.842 | 58.981 | 78.289 | 91.186 | 96.737 | 178.309 | 177.983 | 17.964 | 18.201 | 0.837 | 6.479 | 1.837 | 17.090 | 0.772 | 3.536 |
| C110 | 14.570 | 46.986 | 63.857 | 81.210 | 94.655 | 100.474 | 181.561 | 181.645 | 16.837 | 17.127 | 0.849 | 6.394 | 1.914 | 16.831 | 0.746 | 3.357 |
| C111 | 13.971 | 43.121 | 58.361 | 73.711 | 85.713 | 90.976 | 176.044 | 176.226 | 18.528 | 18.811 | 0.858 | 6.787 | 1.952 | 17.666 | 0.745 | 3.484 |
| C113 | 13.637 | 41.623 | 56.103 | 69.667 | 81.376 | 86.585 | 171.819 | 172.289 | 14.444 | 14.718 | 0.837 | 5.870 | 1.880 | 15.538 | 0.752 | 3.151 |
| C115 | 14.145 | 41.528 | 56.322 | 75.116 | 88.363 | 94.240 | 178.169 | 177.632 | 17.266 | 17.550 | 0.872 | 6.731 | 2.060 | 17.293 | 0.725 | 3.278 |
| C116 | 13.512 | 40.290 | 55.826 | 74.026 | 86.680 | 92.314 | 177.688 | 177.418 | 16.130 | 16.463 | 0.848 | 6.258 | 1.907 | 16.534 | 0.741 | 3.296 |
| C117 | 14.500 | 44.583 | 60.857 | 81.291 | 92.860 | 97.914 | 178.200 | 177.679 | 16.742 | 17.015 | 0.823 | 6.149 | 1.776 | 16.476 | 0.775 | 3.475 |
| C118 | 13.895 | 40.966 | 55.533 | 73.814 | 84.458 | 89.184 | 174.994 | 174.684 | 17.099 | 17.395 | 0.847 | 6.411 | 1.886 | 16.838 | 0.758 | 3.405 |
| C119 | 14.329 | 44.935 | 60.251 | 79.505 | 89.789 | 94.246 | 176.454 | 176.224 | 17.484 | 17.786 | 0.876 | 6.809 | 2.084 | 17.496 | 0.716 | 3.272 |
| C120 | 14.836 | 45.967 | 62.260 | 83.355 | 95.213 | 100.370 | 179.839 | 179.356 | 18.784 | 19.065 | 0.845 | 6.701 | 1.885 | 17.643 | 0.756 | 3.574 |
| C121 | 14.286 | 46.620 | 62.823 | 84.505 | 96.146 | 101.179 | 182.385 | 181.961 | 15.658 | 15.946 | 0.839 | 6.073 | 1.857 | 16.152 | 0.752 | 3.289 |
| C122 | 14.115 | 46.547 | 64.861 | 85.798 | 97.865 | 103.133 | 179.142 | 178.334 | 19.135 | 19.465 | 0.815 | 6.511 | 1.744 | 17.607 | 0.774 | 3.750 |
| C123 | 13.749 | 40.730 | 57.250 | 80.059 | 92.375 | 97.730 | 181.392 | 180.919 | 14.998 | 15.323 | 0.839 | 5.948 | 1.850 | 15.929 | 0.743 | 3.224 |
| C124 | 13.313 | 38.933 | 55.241 | 77.793 | 90.700 | 96.312 | 179.899 | 179.342 | 15.680 | 15.974 | 0.803 | 5.814 | 1.694 | 15.877 | 0.783 | 3.444 |
| C132 | 13.406 | 41.018 | 57.762 | 76.600 | 89.589 | 95.261 | 175.652 | 175.290 | 14.985 | 15.243 | 0.855 | 6.104 | 1.955 | 15.903 | 0.744 | 3.141 |
| C134 | 14.887 | 44.162 | 60.173 | 79.183 | 89.526 | 93.987 | 174.816 | 174.889 | 16.458 | 16.765 | 0.867 | 6.494 | 2.012 | 16.862 | 0.726 | 3.235 |
| C137 | 14.541 | 43.236 | 58.981 | 77.267 | 87.171 | 91.351 | 174.859 | 175.116 | 19.052 | 19.353 | 0.887 | 7.269 | 2.182 | 18.438 | 0.703 | 3.345 |
| C138 | 14.445 | 42.545 | 58.187 | 77.631 | 88.837 | 93.633 | 176.062 | 175.888 | 18.053 | 18.365 | 0.832 | 6.471 | 1.823 | 17.229 | 0.764 | 3.564 |
| C159 | 14.679 | 44.175 | 59.585 | 78.793 | 89.991 | 94.857 | 175.642 | 175.279 | 19.497 | 19.865 | 0.868 | 7.102 | 2.033 | 18.570 | 0.714 | 3.507 |
| C163 | 14.284 | 43.142 | 59.779 | 81.336 | 94.601 | 100.462 | 181.992 | 181.505 | 17.096 | 17.395 | 0.850 | 6.469 | 1.921 | 16.990 | 0.744 | 3.385 |
| C196 | 14.002 | 40.371 | 55.783 | 76.588 | 89.170 | 94.734 | 178.302 | 177.853 | 18.989 | 19.292 | 0.848 | 6.775 | 1.895 | 17.812 | 0.752 | 3.580 |
| C197 | 15.194 | 48.377 | 69.387 | 92.896 | 104.570 | 109.582 | 182.493 | 181.726 | 15.395 | 15.655 | 0.852 | 6.140 | 1.928 | 16.041 | 0.751 | 3.200 |
| C201 | 14.338 | 47.624 | 63.862 | 83.275 | 95.317 | 100.542 | 181.218 | 181.030 | 17.301 | 17.617 | 0.873 | 6.741 | 2.064 | 17.379 | 0.719 | 3.273 |
| C202 | 14.627 | 40.358 | 55.807 | 75.838 | 86.459 | 91.022 | 175.521 | 175.885 | 15.939 | 16.240 | 0.877 | 6.534 | 2.099 | 16.696 | 0.718 | 3.125 |
| DX1 | 13.389 | 41.402 | 58.846 | 78.537 | 91.882 | 97.673 | 178.306 | 178.154 | 15.518 | 15.758 | 0.822 | 5.923 | 1.778 | 15.842 | 0.776 | 3.347 |
| DX2 | 14.599 | 45.643 | 62.456 | 85.161 | 96.824 | 101.812 | 182.150 | 181.559 | 18.222 | 18.544 | 0.832 | 6.490 | 1.818 | 17.353 | 0.760 | 3.582 |

**Supplementary Table S2.** Descriptive statistics of the 16 quality-related traits at the seed level.

| **Trait** | **Mean** | **STD** | **Min** | **25%** | **50%** | **75%** | **Max** | **Heritability** |  |
| --- | --- | --- | --- | --- | --- | --- | --- | --- | --- |
| 375 nm reflectance | 14.116 | 0.672 | 12.255 | 13.697 | 14.126 | 14.541 | 16.749 | 0.251 |  |
| 450 nm reflectance | 42.755 | 2.825 | 34.076 | 40.738 | 42.460 | 44.785 | 51.199 | 0.305 |  |
| 525 nm reflectance | 58.852 | 3.803 | 49.008 | 56.355 | 58.291 | 61.029 | 75.183 | 0.393 |  |
| 630 nm reflectance | 77.908 | 4.952 | 64.669 | 74.826 | 77.739 | 80.834 | 97.556 | 0.482 |  |
| 645 nm reflectance | 89.674 | 5.004 | 73.771 | 86.659 | 89.457 | 92.476 | 110.450 | 0.457 |  |
| 660 nm reflectance | 94.776 | 5.069 | 77.839 | 91.814 | 94.509 | 97.574 | 115.763 | 0.451 |  |
| 940 nm reflectance | 176.963 | 2.896 | 168.119 | 175.018 | 176.817 | 178.986 | 185.438 | 0.217 |  |
| 970 nm reflectance | 176.781 | 2.768 | 168.136 | 174.976 | 176.715 | 178.702 | 184.924 | 0.208 |  |
| Seed area (mm^2^) | 17.047 | 1.605 | 11.471 | 15.997 | 17.117 | 18.085 | 22.707 | 0.250 |  |
| Seed convex area (mm^2^) | 17.348 | 1.613 | 11.695 | 16.299 | 17.437 | 18.386 | 23.088 | 0.248 |  |
| Seed eccentricity | 0.846 | 0.021 | 0.775 | 0.832 | 0.846 | 0.860 | 0.917 | 0.296 |  |
| Seed length (mm) | 6.426 | 0.361 | 5.209 | 6.169 | 6.419 | 6.681 | 7.473 | 0.344 |  |
| Seed LWR | 1.912 | 0.131 | 1.598 | 1.824 | 1.895 | 1.983 | 2.641 | 0.299 |  |
| Seed perimeter (mm) | 16.936 | 0.831 | 13.841 | 16.413 | 16.904 | 17.506 | 19.851 | 0.265 |  |
| Seed roundness | 0.746 | 0.026 | 0.576 | 0.731 | 0.749 | 0.764 | 0.808 | 0.270 |  |
| Seed width (mm) | 3.385 | 0.198 | 2.433 | 3.267 | 3.394 | 3.527 | 3.925 | 0.200 |  |

**Supplementary Figure S1.** The comparison between initial watershed seed segmentation and our improved seed segmentation method.


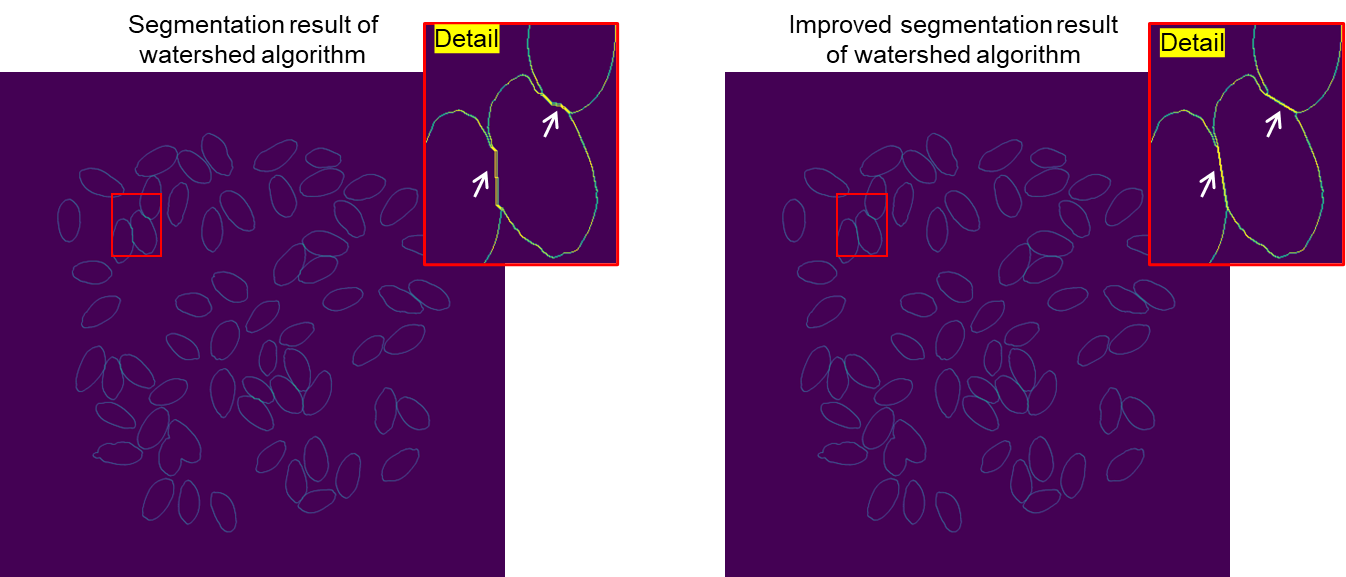


**Supplementary Figure S2.** The correction of over-segmented seed objects.


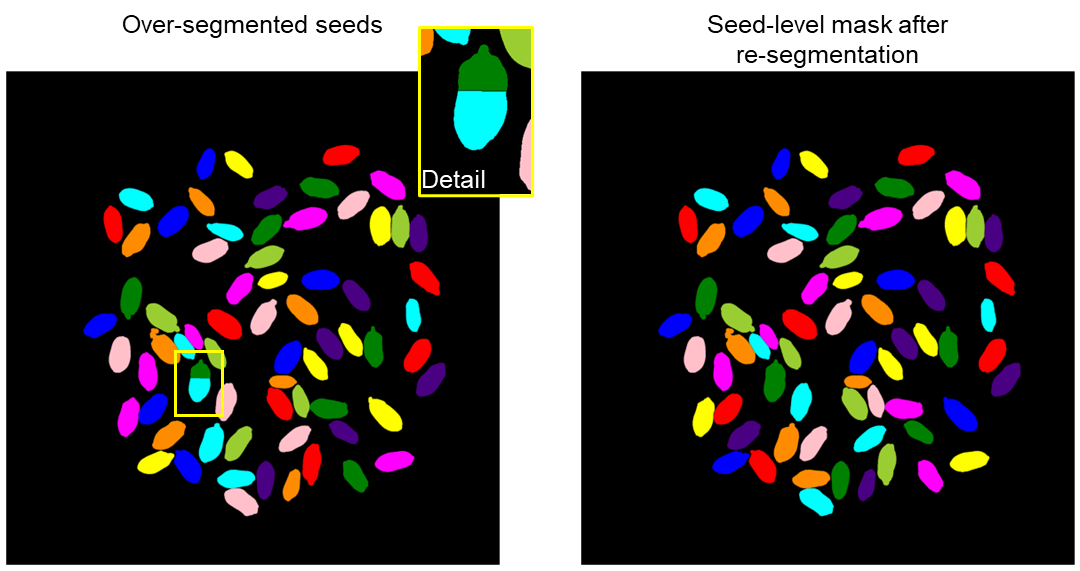


**Supplementary Figure S3.** ADMIXTURE analysis to visualise population structure with K ranging from 3 to 9.


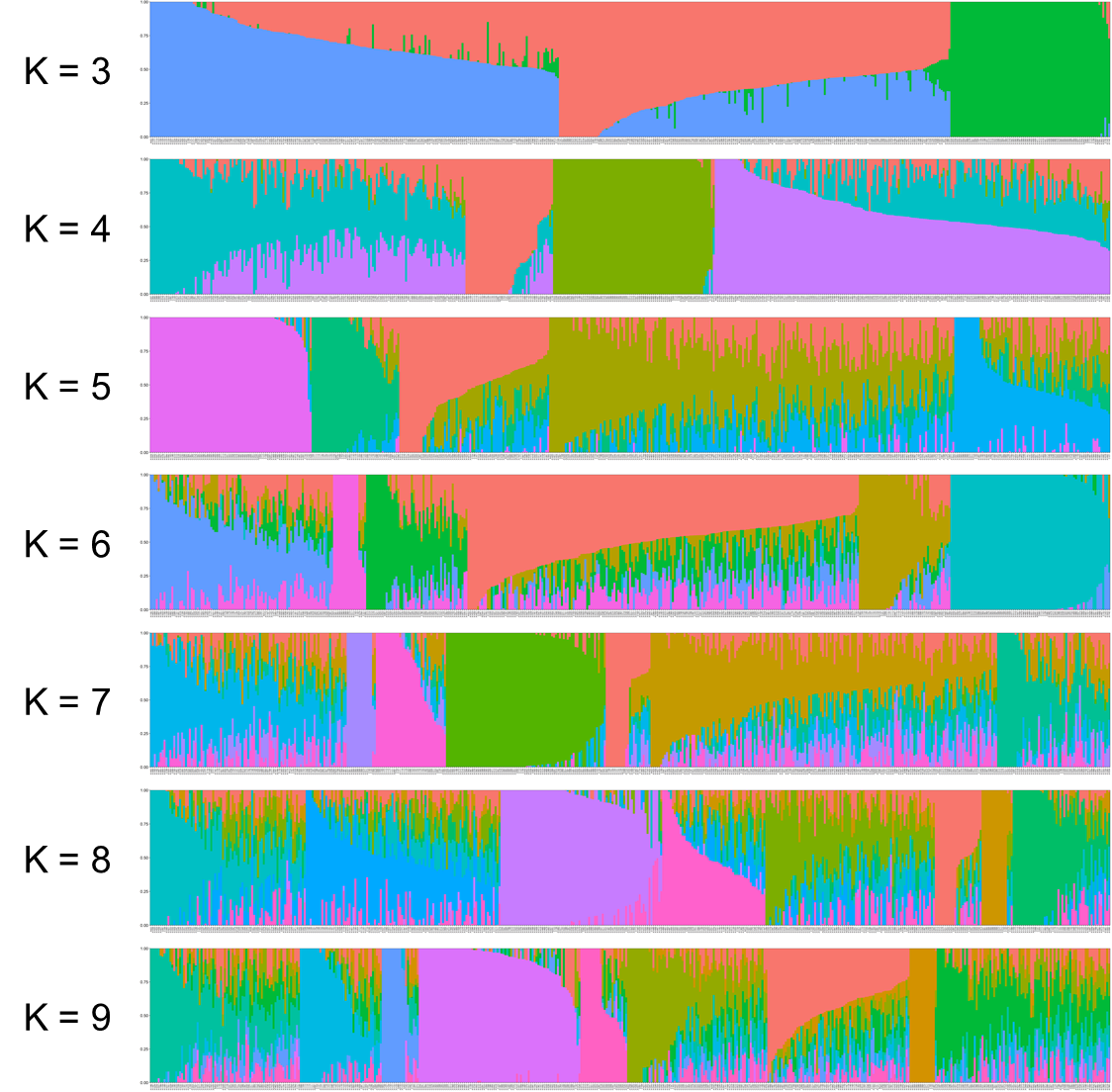


**Supplementary Figure S4.** Correlation analysis between computationally measured and manually scored of seed number from 100 seed lots.


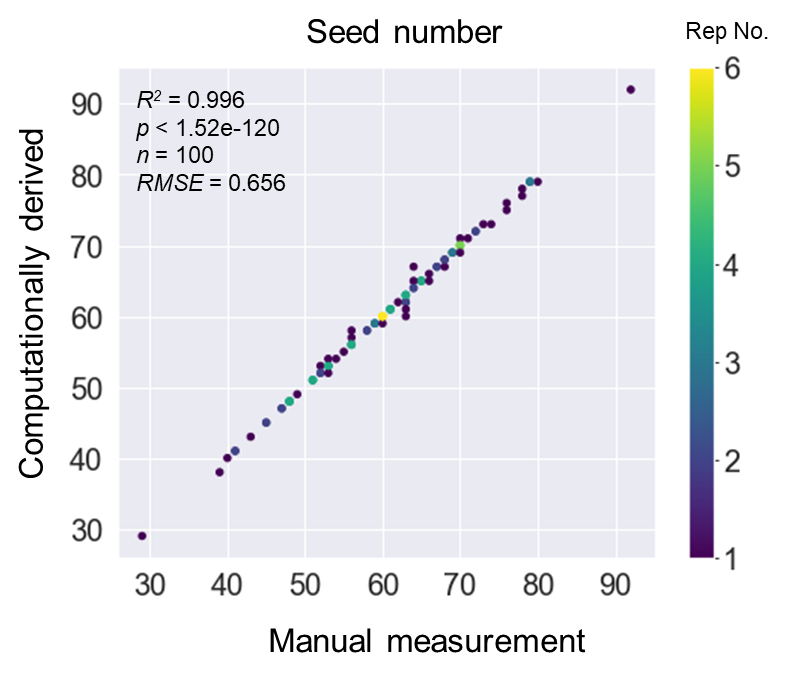


**Supplementary Figure S5.** Frequency distribution and fitted curves of 16 quality-related traits.


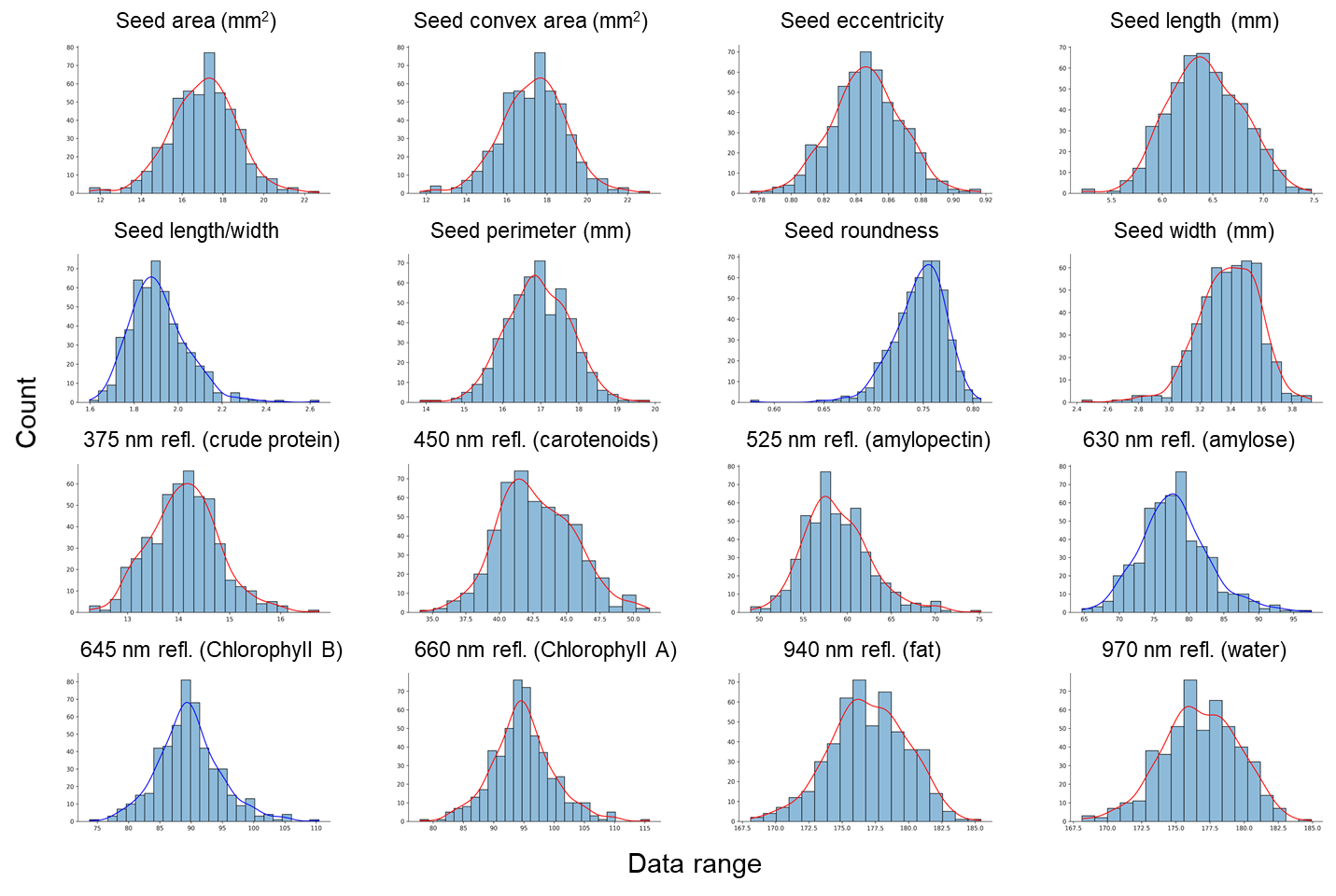


**Note**: the red distribution line indicates that the corresponding trait follows a normal distribution while the blue line does not.
